# Rapid, two-pot procedure for the synthesis of dihydropyridinones; total synthesis of aza-goniothalamin

**DOI:** 10.3762/bjoc.16.15

**Published:** 2020-01-28

**Authors:** Thomas J Cogswell, Craig S Donald, Rodolfo Marquez

**Affiliations:** 1 School of Chemistry, University of Glasgow, Glasgow, G12 8QQ, U.K; 2 Lucideon Limited., Queens Road, Penkhull, Stoke-on-Trent, Staffordshire, ST4 7LQ, U.K; 3 School of Physical and Chemical Sciences, University of Canterbury, Christchurch, 8140, New Zealand

**Keywords:** amidoallylation, aza-goniothalamin, dihydropyridinones, protecting-group-free, ring-closing metathesis, two-pot procedure

## Abstract

A fast, protecting-group-free synthesis of dihydropyridinones has been developed. Starting from commercially available aldehydes, a novel one-pot amidoallylation gave access to diene compounds in good yields. Ring-closing metathesis conditions were then employed to produce the target dihydropyridinones efficiently and in high yields.

## Introduction

Six-membered nitrogen heterocycles are prevalent in many naturally occurring and biologically active compounds. As a result, their synthesis has received extensive research and wide spread publication in the literature [1–6]. Dihydropyridinones are an important subclass of heterocycles, which often feature both as useful intermediates, and as interesting species in their own right [7–13].

**Figure 1:**
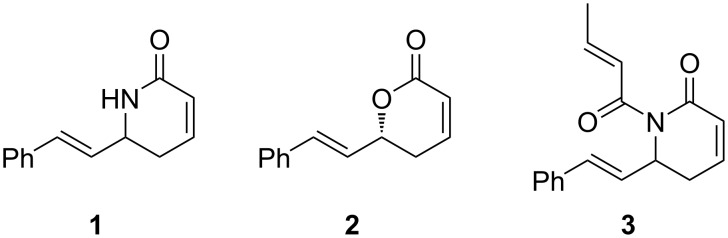
Aza-goniothalamin **1**, (*R*)-(+)-goniothalamin **2** and acylated aza-goniothalamin analogue **3** [14–18].

The relevance of dihydropyridones as lead compounds is exemplified by the detailed investigations into the synthesis and biological evaluation of aza-goniothalamin **1** and its analogues (Figure 1). (*R*)-(+)-Goniothalamin **2**, a natural product isolated in 1967, was shown to be cytotoxic against human leukaemia, kidney, ovarian and prostate cancer cell lines (IC_50_ = 25 µM, U251 cell line) [14–16]. In an attempt to increase the bioavailability of (*R*)-(+)-goniothalamin **2,** aza-goniothalamin **1** was designed and synthesised; however, aza-goniothalamin **1** was found to have significantly lower biological activity compared to the parent compound (IC_50_ = 942 µM, U251 cell line) [15–16]. However, Pilli and co-workers were able to demonstrate that acylation of aza-goniothalamin **1** yielded analogues such as compound **3** with significantly improved biological profiles (IC_50_ = 11 µM, U251 cell line, Figure 1). Thus, a path for the generation of a new class of potential anticancer agents based on the aza-goniothalamin framework was determined [17–18].

## Results and Discussion

**Scheme 1:**
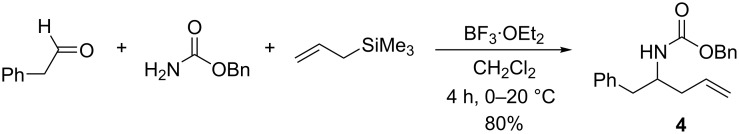
One pot synthesis of benzyl carbamate **4** reported by Veenstra and co-workers [19].

Our approach to the synthesis of the dihydropyridinone framework was inspired by the work carried out by Veenstra and co-workers, who developed a one-pot, three-component reaction to produce protected homoallylic amines **4** (Scheme 1) [19].

It was reasoned that adaptation of the Veenstra protocol would allow us to introduce a second alkene unit during the same process, thus generating a ring-closing metathesis precursor in a single step. This general strategy towards nitrogen heterocycles has been utilized in several reports [20–23], including asymmetric variants [24–25], but in these cases the ring-closing metathesis precursor was always generated in multiple steps.

**Scheme 2:**
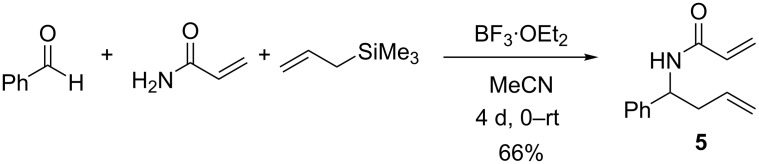
Formation of diene **5** in 66% through a one pot, three component coupling.

In order to test this hypothesis, acrylamide was used in an analogous manner to the carbamates employed by Veenstra (Scheme 1) [19]. In our initial attempts, the solvent was changed from dichloromethane to acetonitrile due to solubility issues with the acrylamide and the postulated imine intermediate. The reaction proved sluggish, taking four days to reach completion; however, the dialkene **5** was isolated in a very encouraging 66% yield (Scheme 2).

**Scheme 3:**
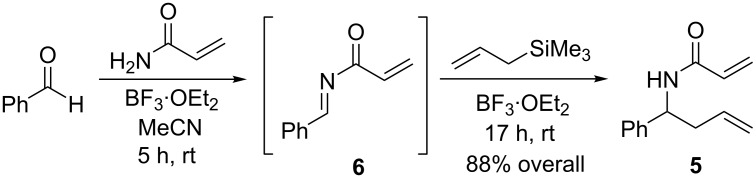
Optimized conditions for the synthesis of diene **5**.

In an attempt to increase the yield of the overall process, the reaction conditions were optimized to reach complete formation of imine **6**, before treatment with allyltrimethylsilane to generate the desired product. This stepwise addition was successful in affording an improved 88% yield of diene **5** as well as significantly reducing the overall reaction time (Scheme 3).

**Scheme 4:**
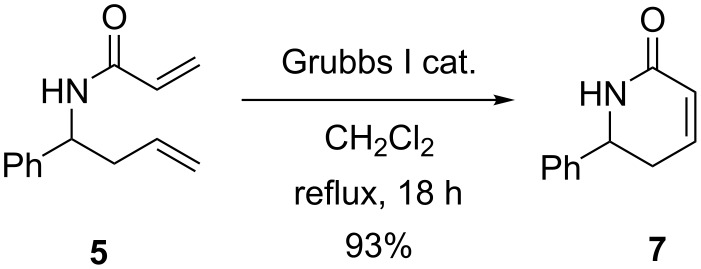
Ring-closing metathesis reaction of diene **5** to yield dihydropyridone **7** [20–23].

Treatment of diene **5** under ring-closing metathesis conditions, using Grubbs I catalyst, then proceeded to generate the target dihydropyridinone **7** in excellent yield (Scheme 4) [20–23,26].

**Figure 2:**
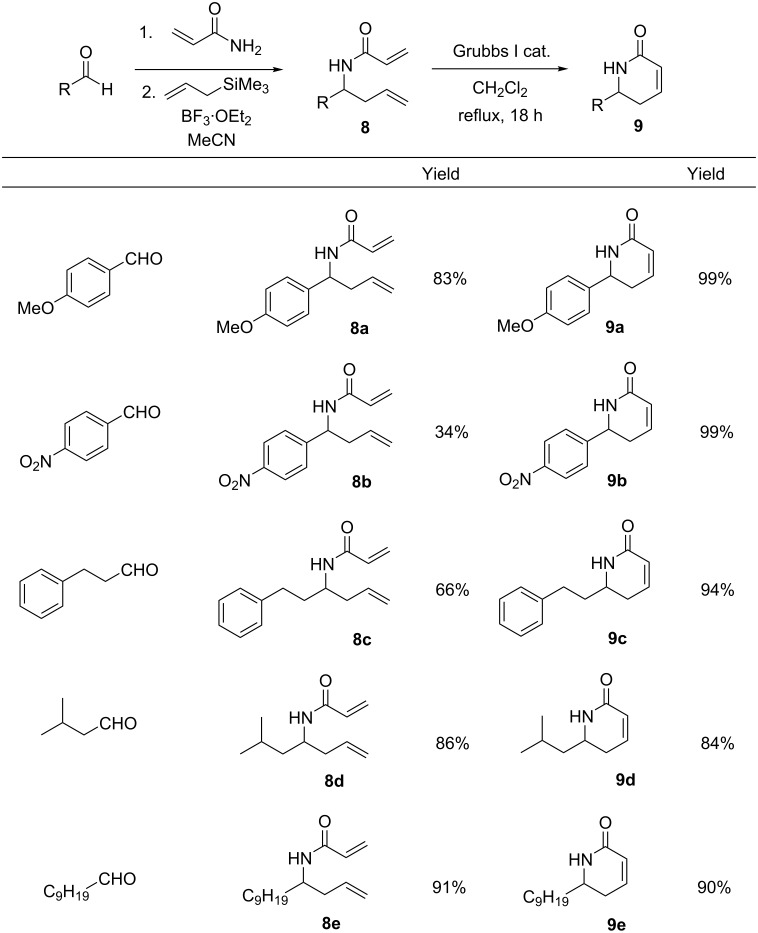
Extension of the two-pot methodology to include a variety of different aldehyde starting materials.

The scope of this concise two-pot methodology was then investigated by using a variety of different aldehydes as starting materials (Figure 2). The electron-donating aromatic substrates and the aliphatic units gave consistently high results over the 2-pot process, producing dihydropyridinones **9a** and **9c**–**e** in high yields. The electron-withdrawing substituted aromatic starting materials on the other hand, gave a low yield in the first step, which we believe was due to poor solubility of the imine intermediate. In contrast, the ring-closing metathesis reaction worked nicely to give **9b** in 99% yield.

**Scheme 5:**
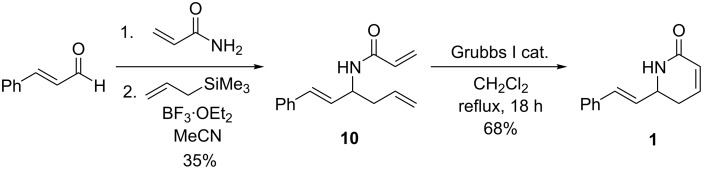
Total synthesis of aza-goniothalamin **1**.

Having demonstrated the versatility of our approach, the synthesis of racemic aza-goniothalamin was attempted. Our synthesis began with cinnamaldehyde (Scheme 5), which was condensed with acrylamide under the same conditions described above. Rewardingly, Hosomi–Sakurai allylation of the conjugated imine intermediate proceeded to afford the desired diene **10** in working yield (35%). The formation of diene **10** is significant as the corresponding α,β-enones and α,β-enals undergo exclusive conjugate addition under Hosomi–Sakurai conditions [27–28]. Ring-closing metathesis of diene **10** then proceeded in good yield (68%) to complete a fast and efficient, protecting-group-free, two-pot procedure for the racemic synthesis of aza-goniothalamin **1**.

## Conclusion

In summary, a two-pot, protecting-group-free procedure for the synthesis of dihydropyridinones has been developed. The process requires a one-pot amidoallylation followed by a ring-closing metathesis step. This approach was used to complete the racemic synthesis of aza-goniothalamin **1**, and is currently being expanded to generate new biologically relevant derivatives.

## Supporting Information

File 1 Experimental, characterization data and copies of spectra.
