# Supplementary material for: Rapid, two-pot procedure for the synthesis of dihydropyridinones; total synthesis of aza-goniothalamin
Source: Beilstein J Org Chem. 2020 Jan 28;16:135–9. doi: 10.3762/bjoc.16.15 (PMC7006497; doi:10.3762/bjoc.16.15)

BEILSTEIN JOURNAL OF ORGANIC CHEMISTRY

Supporting Information

for

Rapid, two-pot procedure for the synthesis of dihydropyridinones; total synthesis of aza-goniothalamin

Thomas J. Cogswell, Craig S. Donald and Rodolfo Marquez

Beilstein J. Org. Chem. **2020,** *16,* 135–139. doi:10.3762/bjoc.16.15

Experimental, characterization data and copies of spectra

License and Terms: This is a supporting information file under the terms of the Creative Commons Attribution License (https://creativecommons.org/licenses/by/4.0). Please note that the reuse, redistribution and reproduction in particular requires that the authors and source are credited.
The license is subject to the *Beilstein Journal of Organic Chemistry* terms and conditions: (https://www.beilstein-journals.org/bjoc)

General

All reactions were performed in oven-dried glassware under an inert argon atmosphere unless otherwise stated. Tetrahydrofuran (THF), diethyl ether, toluene and dichloromethane (DCM) were purified through a Pure Solv 400-5MD solvent purification system (Innovative Technology, Inc). All reagents were used as received, unless otherwise stated. Solvents were evaporated under reduced pressure at 40 °C using a Büchi Rotavapor unless otherwise stated. IR spectra were recorded as thin films on NaCl plates using a JASCO FT/IR410 Fourier Transform spectrometer. Only significant absorptions (ν_{max}) are reported in wavenumbers (cm^{-1}). Proton magnetic resonance spectra (^1H NMR), and carbon magnetic resonance spectra (^{13}C NMR) were respectively recorded at 400 MHz and 100 MHz using a Bruker DPX Avance400 instrument. Proton magnetic resonance spectra (^1H NMR) and carbon magnetic resonance spectra (^{13}C NMR) were respectively recorded at 500 MHz and 125 MHz using a Bruker DPX Avance500 instrument. Chemical shifts (δ) are reported in parts per million (ppm) and are referenced to the residual solvent peak. The order of citation in parentheses is (1) number of equivalent nuclei (by integration), (2) multiplicity (s = singlet, d = doublet, t = triplet, q = quartet, quint = quintet, sext = sextet, sept = septet, m = multiplet, br = broad, appt = apparent), (3) and coupling constant (*J*) quoted in Hertz to the nearest 0.1 Hz. High resolution mass spectra were recorded on a JEOL JMS-700 spectrometer by electrospray (EI) or chemical ionisation (CI) mass spectrometery operating at a resolution of 15000 full widths at half height. Flash chromatography was performed using silica gel (Flurochem Silica Gel 60, 40–63 micron) as the stationary phase. TLC was performed on aluminium sheets pre-coated with silica (Merck Silica Gel 60 F254) unless otherwise stated. The plates were visualised by the quenching of UV fluorescence (λ_{max} 254 nm) and/or by staining with anisaldehyde, potassium permanganate, iodine or cerium ammonium molybdate followed by heating.

General procedure A

A solution of aldehyde (1 equiv) in MeCN (2 mL) was treated with acrylamide (3 equiv) and the resulting solution was cooled down to 0 °C. The solution was then treated slowly with $BF_3{\cdot}OEt_2$ (1 equiv), and the reaction mixture was stirred for 15 min at 0 °C. The solution was then allowed to warm up to rt and then stirred for a further 5 h. At this point, allyltrimethylsilane (2 equiv) and $BF_3{\cdot}OEt_2$ (1–2 equiv) were added, and the resulting mixture was stirred for a further 17 h. The reaction was then poured into sat. aq. $NaHCO_3$ (10 mL), and extracted with CH_2Cl_2 (3 × 20 mL). The combined organic phases were dried (Na_2SO_4), filtered and the solvent was removed in vacuo. The resulting residue was purified by flash column chromatography to yield the desired bis-alkene**.**

General procedure B

A solution of bis-alkene (1 equiv) in CH_2Cl_2 (0.07 M) was treated with Grubbs I catalyst (10 mol %), and the resulting mixture was stirred at reflux until completion as indicated by TLC analysis (17 h). The solvent was removed in vacuo, and the crude residue was purified by flash column chromatography to yield the desired product.

(*E*)-6-Styryl-5,6-dihydropyridin-2(1*H*)-one (1)[1]

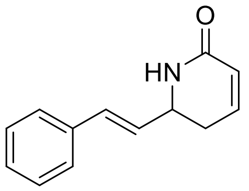

The bis-alkene **10** (80 mg, 0.35 mmol) was subjected to general procedure B. The crude residue was purified by flash column chromatography (silica gel, 15–30% EtOAc in petroleum ether) to yield the desired product **1** as a white solid (50 mg, 0.24 mmol, 68%). ^{1}H NMR ($CDCl_3$, 400 MHz) δ: 7.39-7.29 (5H, m), 6.69-6.59 (2H, m), 6.22 (1H, dd, J_H = 15.6, 7.6 Hz), 5.99 (1H, d, J_H = 8.4 Hz), 5.68 (1H, br s), 4.34-4.32 (1H, m), 2.57-2.55 (1H, m), 2.42-2.39 (1H, m). ^{13}C NMR ($CDCl_3$, 100 MHz) δ: 165.9, 139.8, 135.9, 134.3, 128.8 (2C), 128.5, 128.2, 126.6 (2C), 124.7, 53.7, 30.6.
The spectral data is in agreement with the literature values.[1]

***N*-(1-Phenyl-3-buten-1-yl)propenamide (5)**[2]

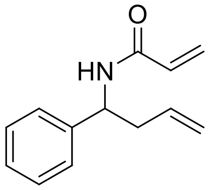

Procedure 1
Benzaldehyde (0.50 g, 4.72 mmol), acrylamide (0.45 g, 7.08 mmol) and allyltrimethylsilane (1.1 mL, 7.08 mmol) were dissolved in MeCN (10 mL). The solution was cooled down to 0 °C before BF_3·OEt_2 (1.1 mL, 8.90 mmol) was added dropwise. The reaction mixture was stirred at 0 °C for 30 min, and then allowed to warm up to rt and stirred for a further 96 h. The reaction mixture was then poured into sat. aq. $NaHCO_3$ (20 mL) and extracted with CH_2Cl_2 (3 × 20 mL). The combined organic extracts were then dried (Na_2SO_4), filtered and the solvent removed in vacuo. The crude residue was purified by flash column chromatography (silica gel, 20% EtOAc in petroleum ether) to yield the desired product **5** as a white solid (0.62 g, 3.11 mmol, 66%).

Procedure 2
Following general procedure A, benzaldehyde (0.20 g, 1.88 mmol) was reacted with acrylamide (0.38 g, 5.66 mmol) and BF_3·OEt_2 (0.23 mL, 1.88 mmol). After imine formation, allyltrimethylsilane (0.59 mL, 3.76 mmol) and BF_3·OEt_2 (0.23 mL, 1.88 mmol) were added. The crude residue was purified by flash column chromatography (silica gel, 20% EtOAc in petroleum ether) to yield the desired bis-alkene **5** as a white solid (0.33 g, 1.65 mmol, 88%) as a pale yellow oil. ^{1}H NMR ($CDCl_3$, 400 MHz) δ: 7.38-7.25 (5H, m), 6.31 (1H, dd, J_H = 16.8, 2.0 Hz), 6.13 (1H, dd, J_H = 16.8, 10.0 Hz), 5.88 (1H, m), 5.78-5.60 (2H,

m), 5.22-5.10 (3H, m), 2.64 (2H, t, J_H = 6.8 Hz). ^{13}C NMR ($CDCl_3$, 125 MHz) δ: 164.7, 141.4, 133.9, 130.8, 128.6 (2C), 127 4, 126.7, 126.5 (2C), 118.3, 52.1, 40.4.
The spectral data is in agreement with the literature values.[2]

6-Phenyl-5,6-dihydro-1*H*-pyridin-2-one (7)[3]

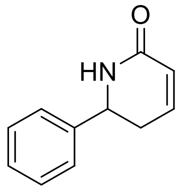

The bis-alkene **5** (0.10 g, 0.50 mmol) was subjected to General Procedure B. The crude residue was purified by flash column chromatography (silica gel, 10–30% EtOAc in petroleum ether) to yield the desired pyridone **7** as a white solid (80 mg, 0.45 mmol, 93%). ^{1}H NMR ($CDCl_3$, 400 MHz) δ: 7.39-7.42 (5H, m), 6.68 (1H, ddd, J_H = 10.0, 5.6, 3.2 Hz), 6.06 (1H, d, J_H = 10.0 Hz), 5.57 (1H, br s) 4.77 (1H, dd, J_H = 10.8, 5.6 Hz), 2.55 (2H, m). ^{13}C NMR ($CDCl_3$, 125 MHz) δ: 166.5, 141.1, 140.2, 129.0 (2C), 128.4, 126.4 (2C), 124.6, 55.9, 33.1.
The spectral data is in agreement with the literature values.[3]

***N*-[1-(4-Methoxyphenyl)-3-buten-1-yl]-propenamide (8a)**[4]

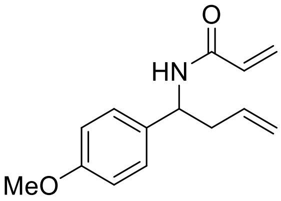

Following general procedure A, 4-methoxybenzaldehyde (0.17 mL, 1.46 mmol) was reacted with acrylamide (0.31 g, 4.38 mmol) and $BF_3 \cdot OEt_2$ (0.17 mL, 1.46 mmol). After imine formation, allyltrimethylsilane (0.46 mL, 2.92 mmol) and $BF_3 \cdot OEt_2$ (0.17 mL, 1.46 mmol) were added. The crude residue was purified by flash column chromatography (silica gel, 20% EtOAc in petroleum ether) to yield the desired product **8a** as a white solid (0.27 g, 1.17 mmol, 83%). ^{1}H NMR ($CDCl_3$, 400 MHz) δ: 7.27 (2H, d, J_H = 8.4 Hz), 6.81 (2H, d, J_H = 8.4 Hz), 6.27 (1H, dd, J_H = 16.8, 1.6 Hz), 6.11 (1H, dd, J_H = 17.2, 10.4 Hz), 5.77-5.71 (1H, m), 5.91 (1H, d, J_H = 7.6 Hz), 5.64 (1H, dd, J_H = 10.4, 1.6 Hz), 5.15-5.10 (3H, m), 3.80 (3H, s), 2.64-2.60 (2H, m). ^{13}C NMR ($CDCl_3$, 125 MHz) δ: 164.7, 158.9, 134.1, 133.6, 130.9, 127.7 (2C), 126.7, 118.1, 114.0 (2C), 55.3, 52.0, 40.3.
The spectral data is in agreement with the literature values.[4]

***N*-[1-(4-Nitrophenyl)-but-3-enyl]-propenamide (8b)**

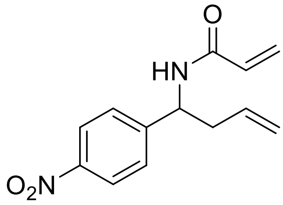

Following general procedure A, 4-nitrobenzaldehyde (0.20 g, 1.3 mmol) was reacted with acrylamide (0.28 g, 4.0 mmol) and $BF_3 \cdot OEt_2$ (0.16 mL, 1.3 mmol). After imine formation, allyltrimethylsilane (0.41 mL, 2.64 mmol) and $BF_3 \cdot OEt_2$ (0.40 mL, 3.4 mmol) were added. The crude residue was purified by flash column chromatography (silica gel, 20% EtOAc in petroleum ether) to yield the desired bis-alkene **8b** as a white solid (0.11 g, 0.44 mmol, 34%). ^{1}H NMR ($CDCl_3$, 400 MHz) δ: 8.11 (2H, d, J_H = 8.5 Hz), 7.38 (2H, d, J_H = 8.5 Hz) 6.38 (1H, dd, J_H = 16.5, 1.0 Hz), 6.14 (1H, d, J_H = 7.0 Hz), 6.08 (1H, dd, J_H = 16.5, 10.0 Hz), 5.63-5.53 (2H, m), 5.13-5.07 (3H, m), 2.53-2.50 (2H, m). ^{13}C NMR ($CDCl_3$, 125 MHz) δ: 165.0, 149.1, 147.2, 132.7, 130.1, 127.6, 127.3 (2C), 123.9 (2C), 119.5, 52.3, 40.2. *m/z* [EI (+ve)] 246.2 $[M]^+$, HRMS found $[M]^+$ 246.1003, $C_{13}H_{14}N_2O_3$ requires 246.1004. IR (thin film) ν_{max} = 3290, 3090, 2920, 1680, 1610, 1525, 1510 cm^{-1}. m.p. 84-86 ˚C.

***N*-(1-Phenethyl-but-3-enyl)-propenamide (8c)**

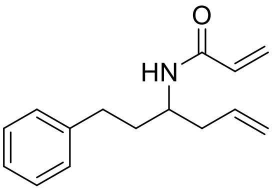

Following general procedure A, 3-phenylpropionaldehyde (0.20 mL, 1.49 mmol) was reacted with acrylamide (0.32 g, 4.47 mmol) and $BF_3 \cdot OEt_2$ (0.18 mL, 1.49 mmol). After imine formation, allyltrimethylsilane (0.47 mL, 2.98 mmol) and $BF_3 \cdot OEt_2$ (0.18 mL, 1.78 mmol) were added. The crude residue was purified by flash column chromatography (silica gel, 10% EtOAc in petroleum ether) to yield the desired bis-alkene **8c** as a white solid (0.22 g, 1.00 mmol, 66%). ^{1}H NMR ($CDCl_3$, 400 MHz) δ: 7.32-7.19 (5H, m), 6.31 (1H, dd, J_H = 17.2, 1.2 Hz), 6.08 (1H, dd, J_H = 17.2, 10.4 Hz), 5.85-5.75 (1H, m), 5.66 (1H, dd, J_H = 10.4, 1.6 Hz), 5.53 (1H, d, J_H = 8.4 Hz), 5.13-5.09 (2H, m), 4.17-4.15 (1H, m), 2.70 (2H, t, J_H = 8.4 Hz) 2.35-2.31 (2H, m), 1.86-1.83 (2H, m). ^{13}C NMR ($CDCl_3$, 125 MHz) δ: 164.7, 141.7, 134.0, 131.0, 128.5 (2C), 128.4 (2C), 126.4, 125.9, 118.2, 48.6, 39.2, 36.2, 32.4. *m/z* [EI (+ve)] 229.2 $[M]^+$, HRMS found $[M]^+$ 229.1465, $C_{15}H_{19}NO$ requires 229.1467. IR (thin film) ν_{max} = 3250, 3100, 2920, 1640, 1605, 1550 cm^{-1}. m.p. 85-87 ˚C.

***N*-(1-Isobutyl-but-3-enyl)-propenamide (8d)**[5]

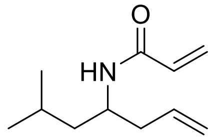

Following general procedure A, isovaleraldehyde (0.25 mL, 2.33 mmol) was reacted with acrylamide (0.49 g, 6.99 mmol) and $BF_3{\cdot}OEt_2$ (0.28 mL, 2.33 mmol). After imine formation, allyltrimethylsilane (0.72 mL, 4.66 mmol) and $BF_3{\cdot}OEt_2$ (0.28 mL, 2.33 mmol) were added. The crude residue was purified by flash column chromatography (silica gel, 20% EtOAc in petroleum ether) to yield the desired bis-alkene **8d** as a white solid (0.36 g, 2.00 mmol, 86%). ^{1}H NMR ($CDCl_3$, 400 MHz) δ: 6.29 (1H, dd, J_H = 17.2, 1.6 Hz), 6.06 (1H, dd, J_H = 17.2, 10.4 Hz), 5.85-5.74 (1H, m), 5.63 (1H, dd, J_H = 10.4, 1.6 Hz), 6.15-6.13 (1H, m), 5.08-5.06 (2H, m), 4.19-4.17 (1H, m), 2.31-2.28 (2H, m), 1.66-1.64 (1H, m), 1.35-1.32 (2H, m), 0.98 (3H, d, J_H = 6.6 Hz), 0.96 (3H, d, J_H = 6.6 Hz). ^{13}C NMR ($CDCl_3$, 125 MHz) δ: 165.0, 134.3, 131.1, 127.6, 117.8, 46.8, 43.7, 39.7, 24.9, 23.1, 23.1.
The spectral data is in agreement with the literature values.[5]

***N*-(1-Allyl-decyl)-propenamide (8e)**

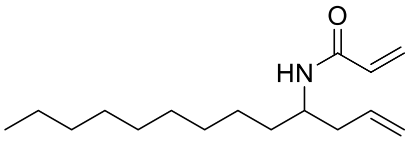

Following general procedure A, decanal (0.24 mL, 1.28 mmol) was reacted with acrylamide (0.27 g, 3.84 mmol) and $BF_3{\cdot}OEt_2$ (0.15 mL, 1.28 mmol). After imine formation, allyltrimethylsilane (0.40 mL, 2.56 mmol) and $BF_3{\cdot}OEt_2$ (0.15 mL, 1.28 mmol) were added. The crude residue was purified by flash column chromatography (silica gel, 5% EtOAc in petroleum ether) to yield the desired bis-alkene **8e** as a white solid (0.29 g, 1.19 mmol, 91%). ^{1}H NMR ($CDCl_3$, 400 MHz) δ: 6.18 (1H, dd, J_H = 16.8, 1.6 Hz), 5.98 (1H, dd, J_H = 16.8, 10.4 Hz), 5.75-5.64 (1H, m), 5.54 (1H, dd, J_H = 10.4, 1.6 Hz), 5.26 (1H, d, J_H = 8.8 Hz), 5.01-4.96 (2H, m), 4.02-4.01 (1H, m), 2.23-2.20 (2H, m), 1.34-1.31 (2H, m), 1.19-1.08 (14H, m), 0.84-0.80 (3H, m). ^{13}C NMR ($CDCl_3$, 100 MHz) δ: 165.0, 134.3, 131.1, 126.1, 117.9, 48.7, 39.1, 34.4, 31.9, 29.5, 29.5, 29.3, 25.9, 25.5, 22.7, 14.1. *m/z* [EI (+ve)] 251.2 $[M]^+$, HRMS found $[M]^+$ 251.2244, $C_{16}H_{29}NO$ requires 251.2249. IR (thin film) ν_{max} = 3267, 2924, 2854, 1654, 1548, 1263, 1099 cm^{-1}. m.p. 56-58 ˚C.

5,6-Dihydro-6-(4-methoxyphenyl)pyridin-2(1*H*)-one (9a)[4]

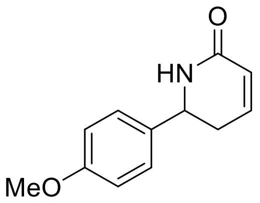

The bis-alkene **8a** (0.10 g, 0.43 mmol) was subjected to general procedure B. The crude residue was purified by flash column chromatography (silica gel, 10–30% EtOAc in petroleum ether) to yield the desired pyridone **9a** as a white solid (90 mg, 0.43 mmol, quantitative yield). ^{1}H NMR ($CDCl_3$, 400 MHz) δ: 7.31 (2H, d, J_H = 8.8 Hz), 6.93 (2H, d, J_H = 8.8 Hz), 6.67 (1H, ddd, J_H = 10.0, 5.2, 3.6 Hz), 6.03 (1H, d, J_H = 10.0 Hz), 5.50 (1H, br s), 4.70 (1H, dd, J_H = 10.0, 6.4 Hz), 3.84 (3H, s), 2.52 (2H, m). ^{13}C NMR ($CDCl_3$, 125 MHz) δ: 166.5, 159.6, 140.3, 133.1, 127.7 (2C), 124.6, 114.3 (2C), 55.4, 55.3, 33.2.
The spectral data is in agreement with the literature values.[4]

5,6-Dihydro-6-(4-nitrophenyl)pyridin-2(1*H*)-one (9b)[6]

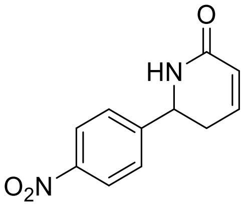

The bis-alkene **8b** (0.10 g, 0.40 mmol) was subjected to general procedure B. The crude residue was purified by flash column chromatography (silica gel, 15–40% EtOAc in petroleum ether) to yield the desired pyridone **9b** as a white solid (90 mg, 0.40 mmol, quantitative yield). ^{1}H NMR ($CDCl_3$, 400 MHz) δ: 8.17 (2H, d, J_H = 8.8 Hz), 7.48 (2H, d, J_H = 8.8 Hz), 6.55 (1H, m), 6.15 (1H, br s), 5.96 (1H, d, J_H = 8.0 Hz), 4.82 (1H, dd, J_H = 8.0, 4.8 Hz), 2.63 (1H, m), 2.43 (1H, m).
^{13}C NMR ($CDCl_3$, 125 MHz) δ: 166.3, 148.4, 147.9, 139.5, 127.3 (2C), 124.8, 124.3 (2C), 55.0, 32.6.
The spectral data is in agreement with the literature values.[6]

5,6-Dihydro-6-(phenethyl)pyridin-2(1*H*)-one (9c)

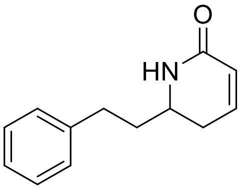

The bis-alkene **8c** (0.10 g, 0.44 mmol) was subjected to general procedure B. The crude residue was purified by flash column chromatography (silica gel, 15–30% EtOAc in petroleum ether) to yield the desired pyridone **9c** as a white solid (80 mg, 0.41 mmol, 94%). ^{1}H NMR ($CDCl_3$, 400 MHz) δ: 7.34-7.20 (5H, m), 6.24-6.21 (1H, m), 5.93 (1H, dd, J_H = 9.6, 1.2 Hz), 5.84 (1H, br s), 3.63-3.62 (1H, m), 2.74-2.70 (2H, m), 2.44-2.41 (1H, m), 2.26-2.24 (1H, m), 1.91-1.89 (2H, m). ^{13}C NMR ($CDCl_3$, 125 MHz) δ: 166.1, 140.6, 140.1, 128.6 (2C), 128.2, 126.3 (2C), 124.6, 50.5, 37.1, 31.6, 29.9. *m/z* [EI (+ve)] 201.2 $[M]^+$, HRMS found $[M]^+$ 201.1153, $C_{13}H_{15}NO$ requires 201.1154. IR (thin film) ν_{max} = 3059, 2922, 2868, 1676, 1608, 1415, 1325 cm^{-1}. m.p. 116-118 ˚C.

5,6-Dihydro-6-(isobutyl)pyridin-2(1*H*)-one (9d)[5]

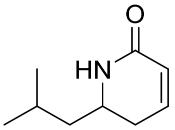

The bis-alkene **8d** (0.12 g, 0.67 mmol) was subjected to general procedure B. The crude residue was purified by flash column chromatography (silica gel, 15–20% EtOAc in petroleum ether) to yield the desired pyridone **9d** as a white solid (90 mg, 0.56 mmol, 84%). ^{1}H NMR ($CDCl_3$, 400 MHz) δ: 6.62 (1H, m), 5.92 (1H, d, J_H = 8.8 Hz), 5.61 (1H, br s), 3.70-3.69 (1H, m), 2.38-2.36 (1H, m), 2.16-2.14 (1H, m), 1.71 (1H, quint, J_H = 6.8 Hz), 1.45-1.43 (2H, m), 0.96 (3H, d, J_H = 6.6 Hz), 0.94 (3H, d, J_H = 6.6 Hz). ^{13}C NMR ($CDCl_3$, 125 MHz) δ: 165.0, 140.5, 124.8, 49.1, 44.8, 30.5, 24.3, 22.7, 22.2. The spectral data is in agreement with the literature values.[5]

5,6-Dihydro-6-(*n*-nonyl)pyridin-2(1*H*)-one (9e)

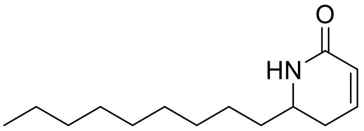

The bis-alkene **8e** (0.10 g, 0.40 mmol) was subjected to general procedure B. The crude residue was purified by flash column chromatography (silica gel, 15–20% EtOAc in petroleum ether) to yield the desired pyridone **9e** as a white solid (80 mg, 0.35 mmol, 90%). ^{1}H NMR ($CDCl_3$, 400 MHz) δ: 6.53 (1H, m), 5.83 (1H, dd, J_H = 8.0, 0.8 Hz), 5.54 (1H, br s), 3.50 (1H, sept, J_H = 4.8 Hz), 2.33-2.30 (1H, m), 2.08-2.06 (1H, m), 1.44-1.41 (2H, m), 1.23-1.18 (14H, m), 0.81 (3H, t, J_H = 5.6 Hz).^{13}C NMR ($CDCl_3$, 125 MHz) δ: 166.5, 140.7, 124.5, 51.1, 35.5, 31.9, 30.0, 29.5, 29.4, 29.3, 25.3, 25.2, 22.7, 14.1.*m/z* [EI (+ve)] 223.3 $[M]^+$, HRMS found $[M]^+$ 223.1933, $C_{14}H_{25}NO$ requires 223.1936. IR (thin film) ν_{max} = 2924, 2852, 2360, 1678, 1610, 1419, 1309 cm^{-1}.m.p. 38-40 ˚C.

(*E*)-*N*-(1-Phenylhexa-1,5-dien-3-yl)acrylamide (10)[1]

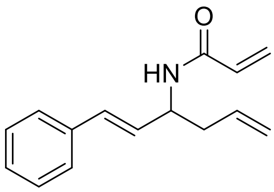

Following general procedure A, cinnamaldehyde (0.24 mL, 1.9 mmol) was reacted with acrylamide (0.40 g, 5.7 mmol) and $BF_3 \cdot OEt_2$ (0.24 mL, 1.9 mmol). After imine formation, allyltrimethylsilane (0.60 mL, 3.9 mmol) and $BF_3 \cdot OEt_2$ (0.48 mL, 3.8 mmol) were added. The crude residue was purified by flash column chromatography (silica gel, 15% EtOAc in petroleum ether) to yield the desired bis-alkene **10** as a white solid (0.15 mg, 0.66 mmol, 35%). ^{1}H NMR ($CDCl_3$, 400 MHz) δ: 7.27-7.11 (5H, m), 6.45 (1H, dd, J_H = 16.8, 1.6 Hz), 6.24 (1H, dd, J_H =10.8, 1.6 Hz), 6.12 (1H, dd, J_H =16.8, 6.5 Hz), 6.03 (1H, d, J_H = 10.8 Hz), 5.75-5.70 (2H, m), 5.59 (1H, dd, J_H = 6.5, 1.6 Hz), 5.14-5.08 (2H, m), 4.76-4.74 (1H, m), 2.41-2.37 (2H, m). ^{13}C NMR ($CDCl_3$, 125 MHz) δ: 164.8, 136.6, 133.7, 130.9 (2C), 128.9, 128.6 (2C), 127.7, 126.7, 126.4 (2C), 118.6, 50.0, 39.4.

The spectral data is in agreement with the literature values.[1]

References

1. Kasaplar, P.; Yilmazar, O.; Çağir, A. *Bioorg. Med. Chem*. **2009**, *17*, 311; Barcelos, R. C.; Pastre, J. C.; Caixeta, V.; Vendramini-Costa, D. B.; de Carvalho, J. E.; Pilli, R. A. *Bioorg. Med. Chem*. **2012**, *20*, 3635
2. Vankar, Y. D.; Kumaravel G.; Rao, C. T. *Syn. Commun*. **1989**, *19*, 2181.
3. Marson, C. M.; Grabowska, U.; Fallah, A.; Walsgrove, T.; Eggleston, D. S.; Baures, P. W. *J. Org. Chem*. **1994** , *59*, 2, 291.
4. Fiorelli, C.; Savoia, D. *J. Org. Chem*. **2007**, *72*, 6022.
5. Lucchetti, N.; Lancianesi, S.; Petrini, M. *Eur. J. Org. Chem.* **2014**, *25* , 5433.
6. Piao, F.; Mishra, M. K.; Jang, D. O. *Tetrahedron*, **2012** , *68*, 7050.

(*E*)-6-Styryl-5,6-dihydropyridin-2(1*H*)-one (1)

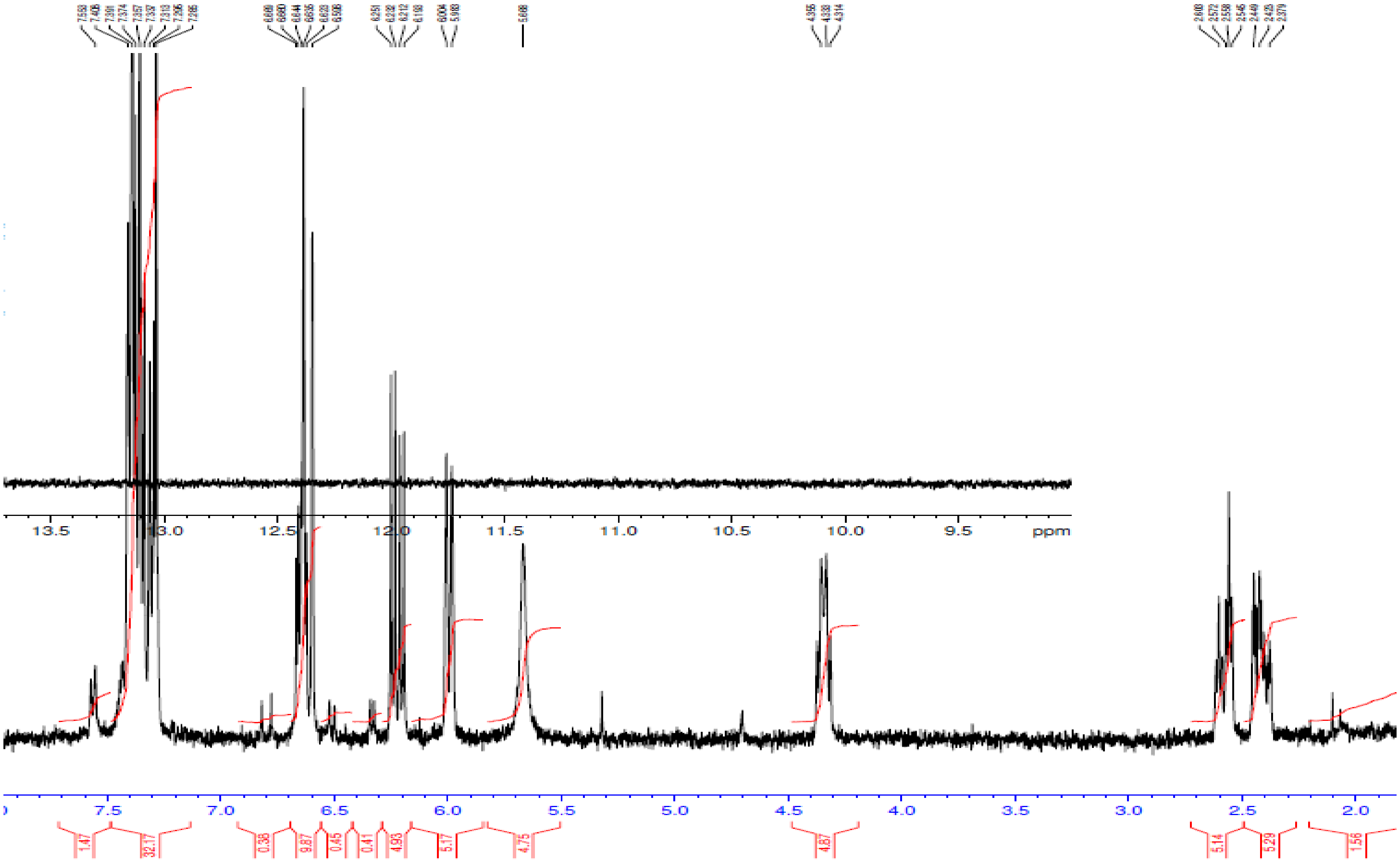

(*E*)-6-Styryl-5,6-dihydropyridin-2(1*H*)-one (1)

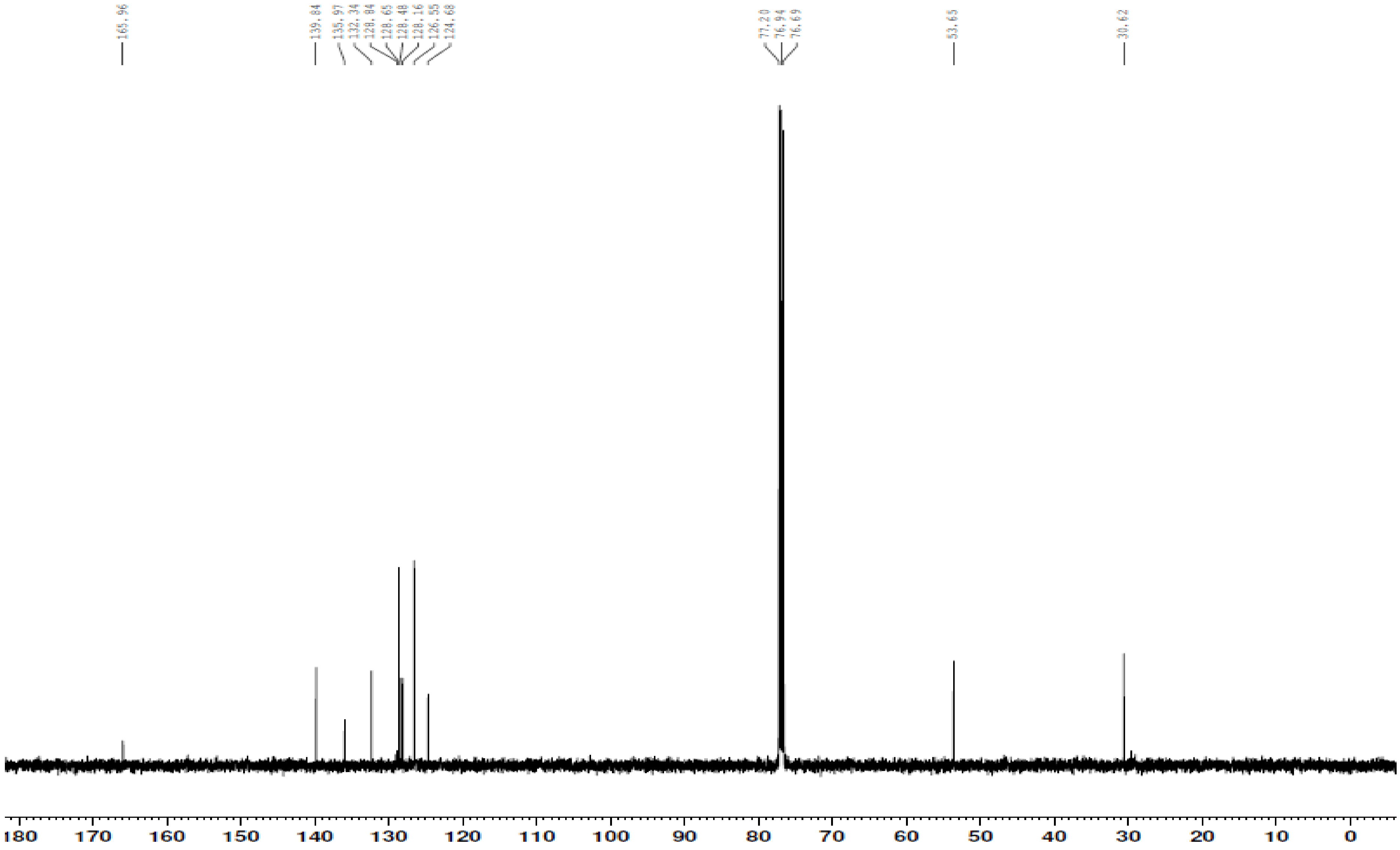

***N*-(1-Phenyl-3-buten-1-yl)propenamide (5)**

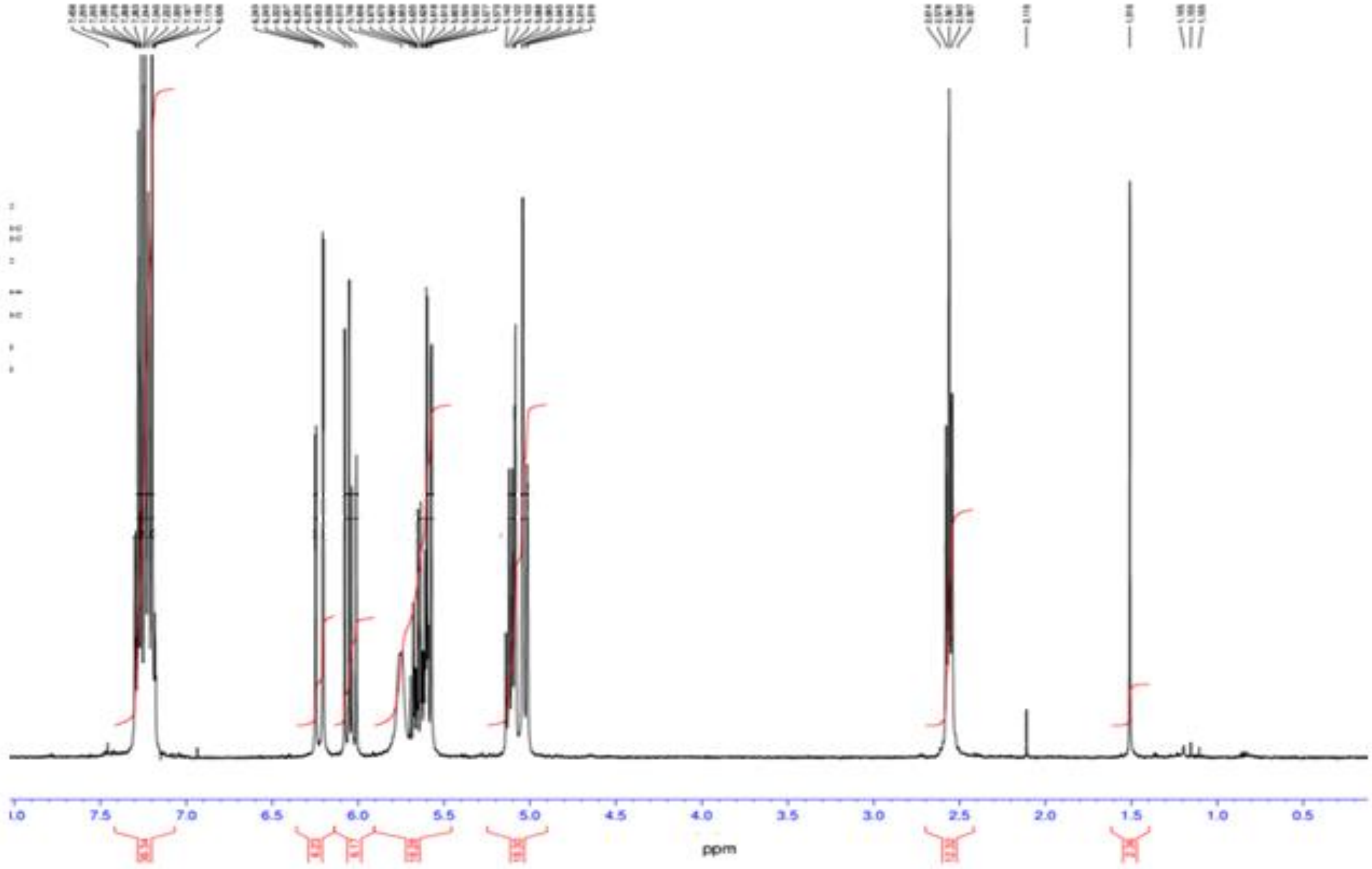

***N*-(1-Phenyl-3-buten-1-yl)propenamide (5)**

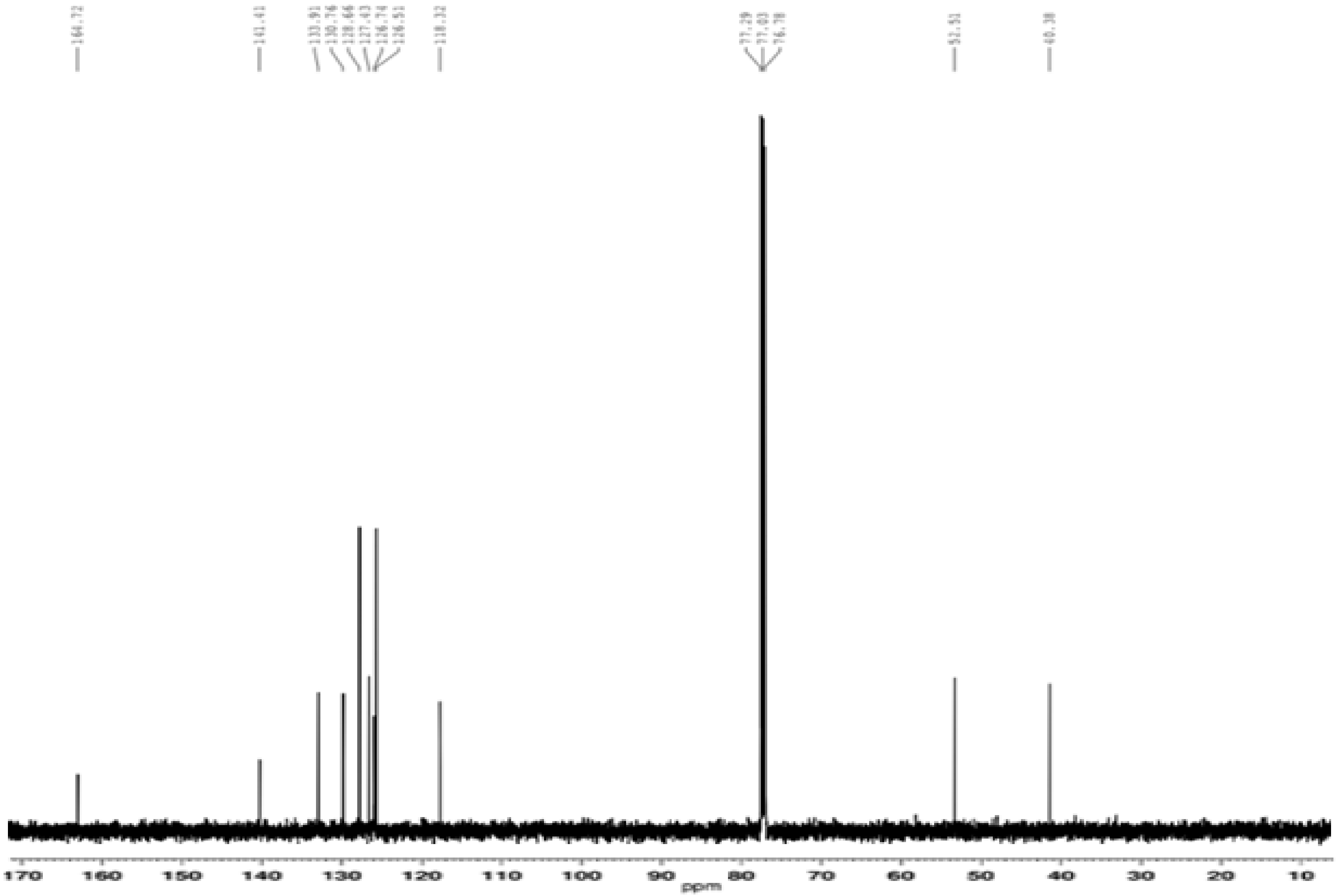

6-Phenyl-5,6-dihydro-1*H*-pyridin-2-one (7)

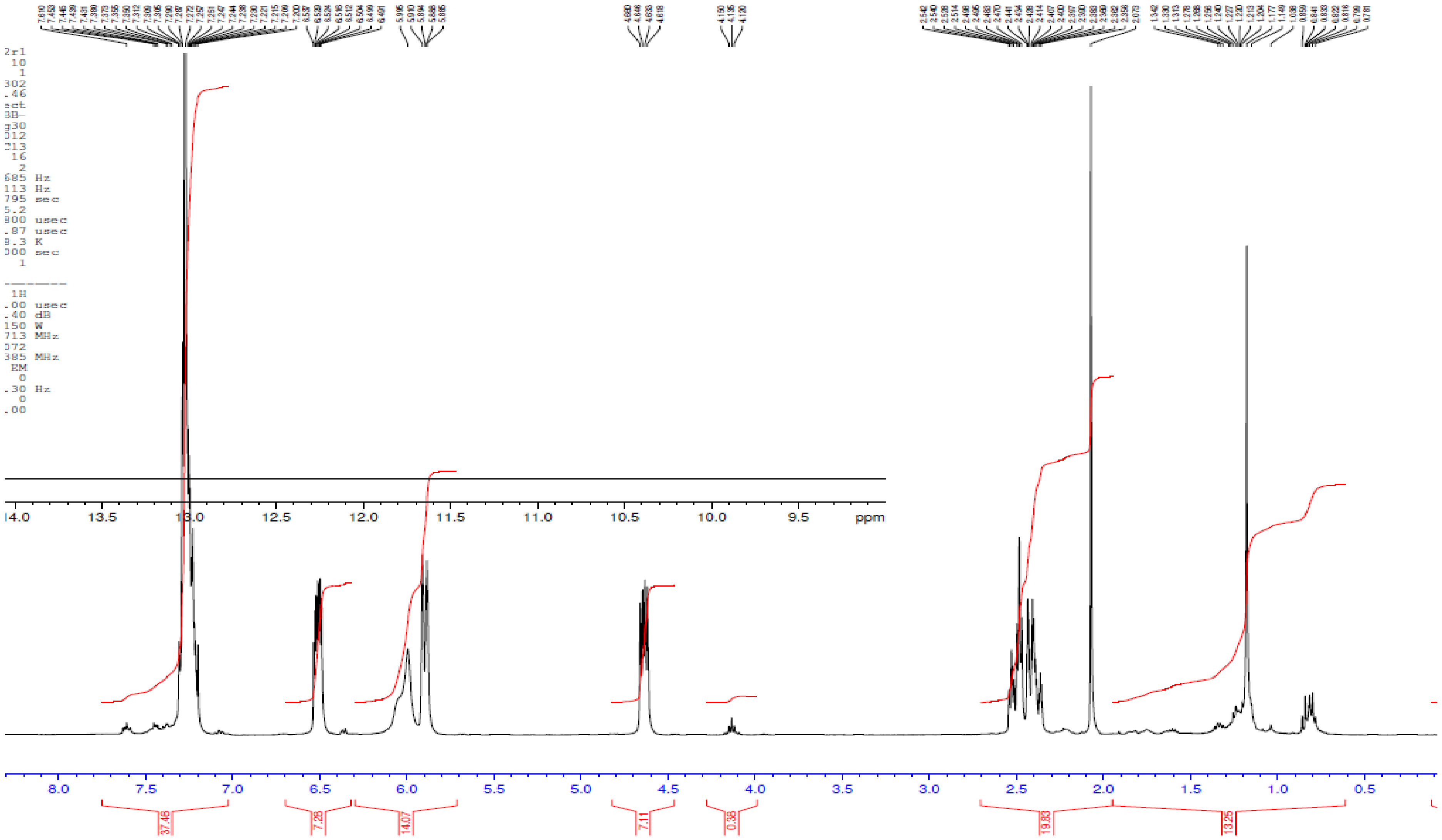

6-Phenyl-5,6-dihydro-1*H*-pyridin-2-one (7)

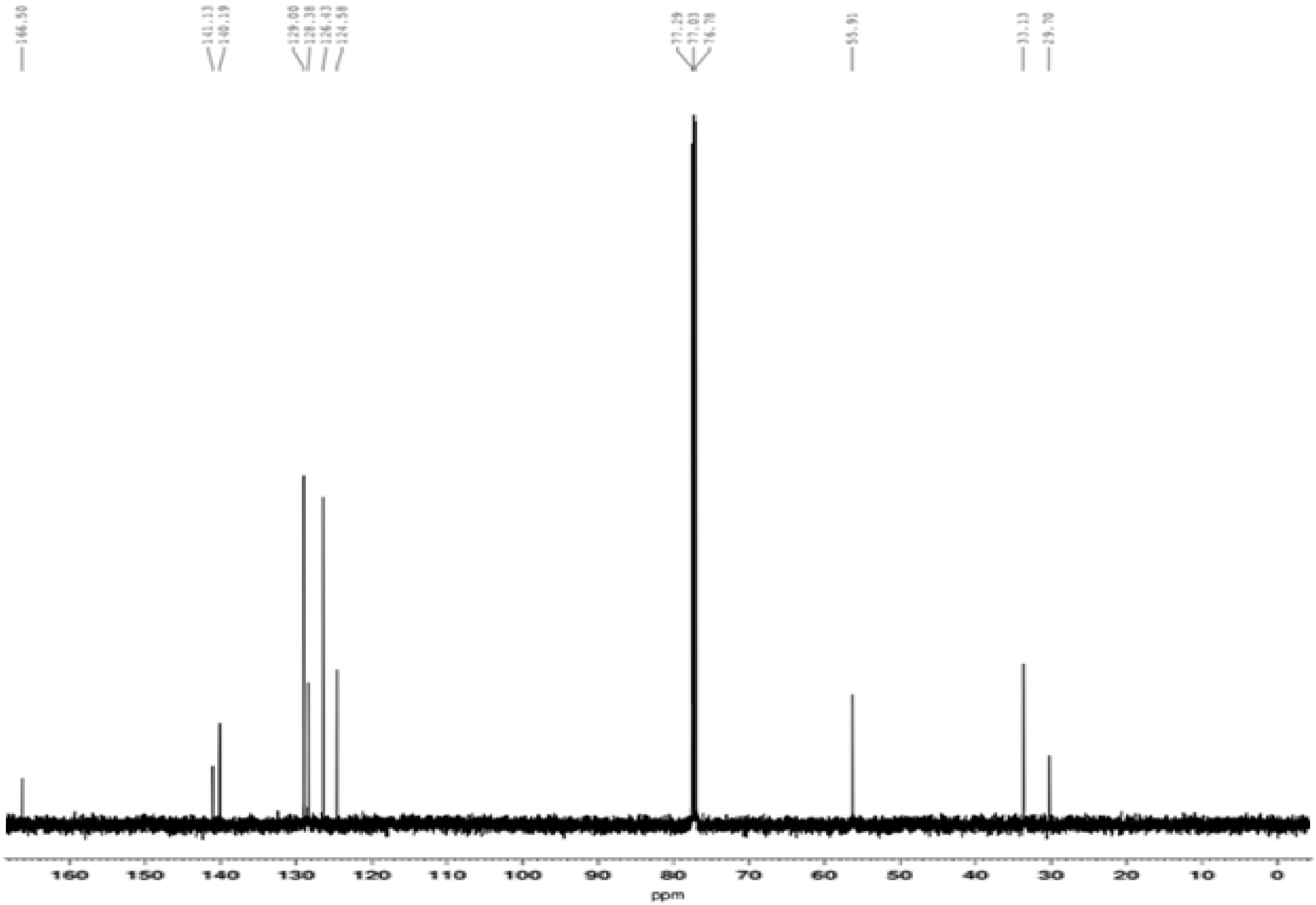

_N_-[1-(4-Methoxyphenyl)-3-buten-1-yl]propenamide (8a)

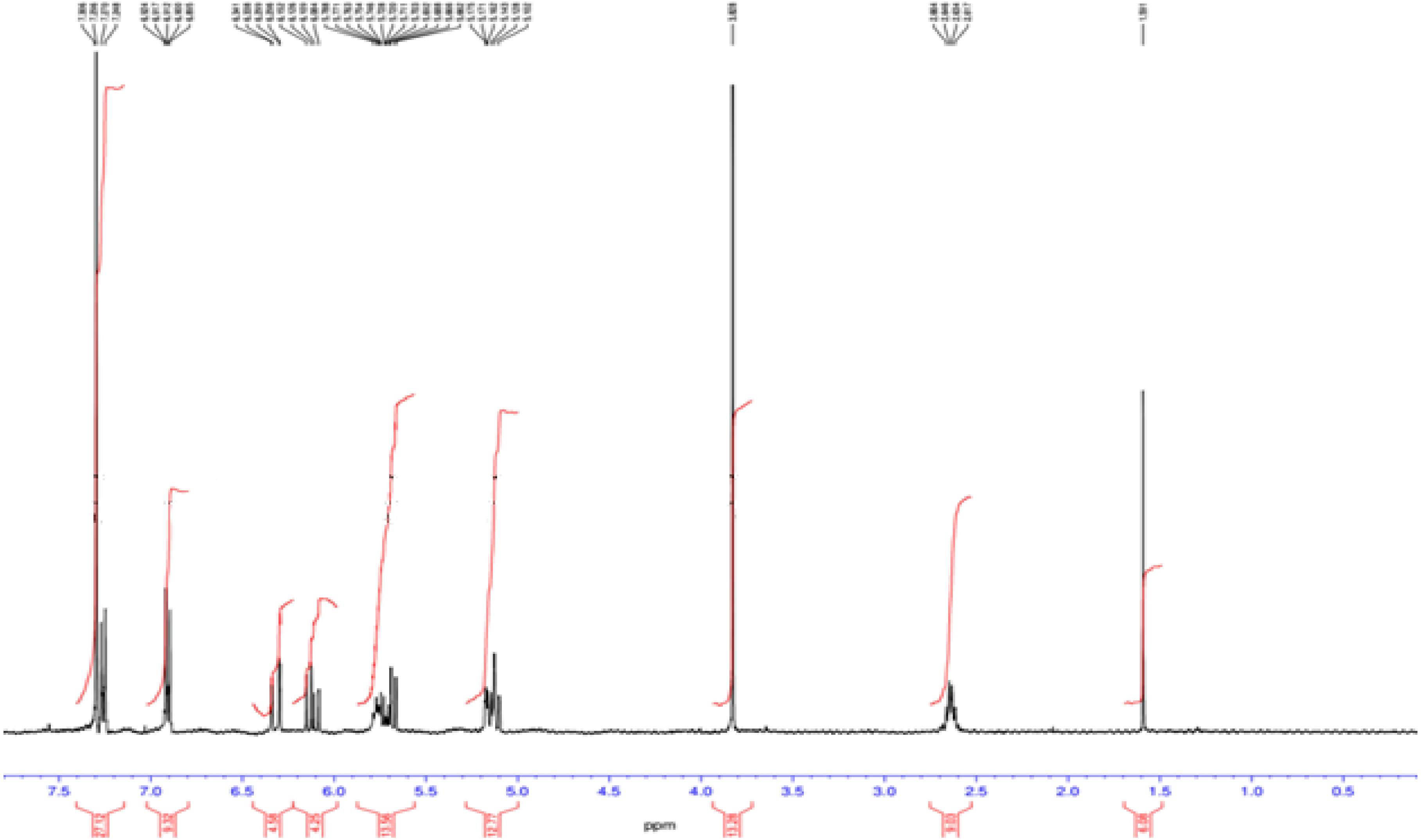

***N*-[1-(4-Methoxyphenyl)-3-buten-1-yl]propenamide (8a)**

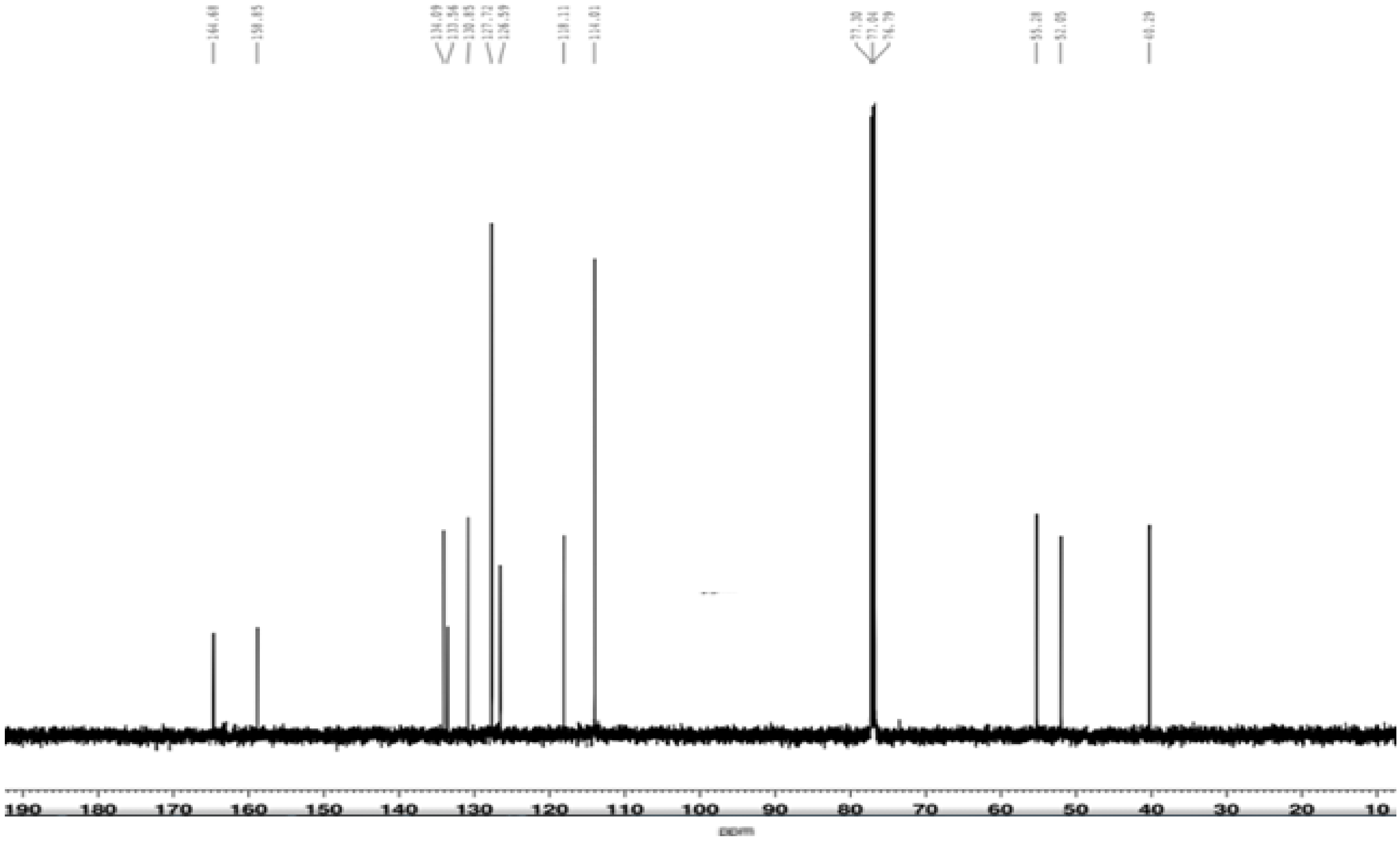

N-[1-(4-Nitrophenyl)-but-3-enyl]propenamide (8b)

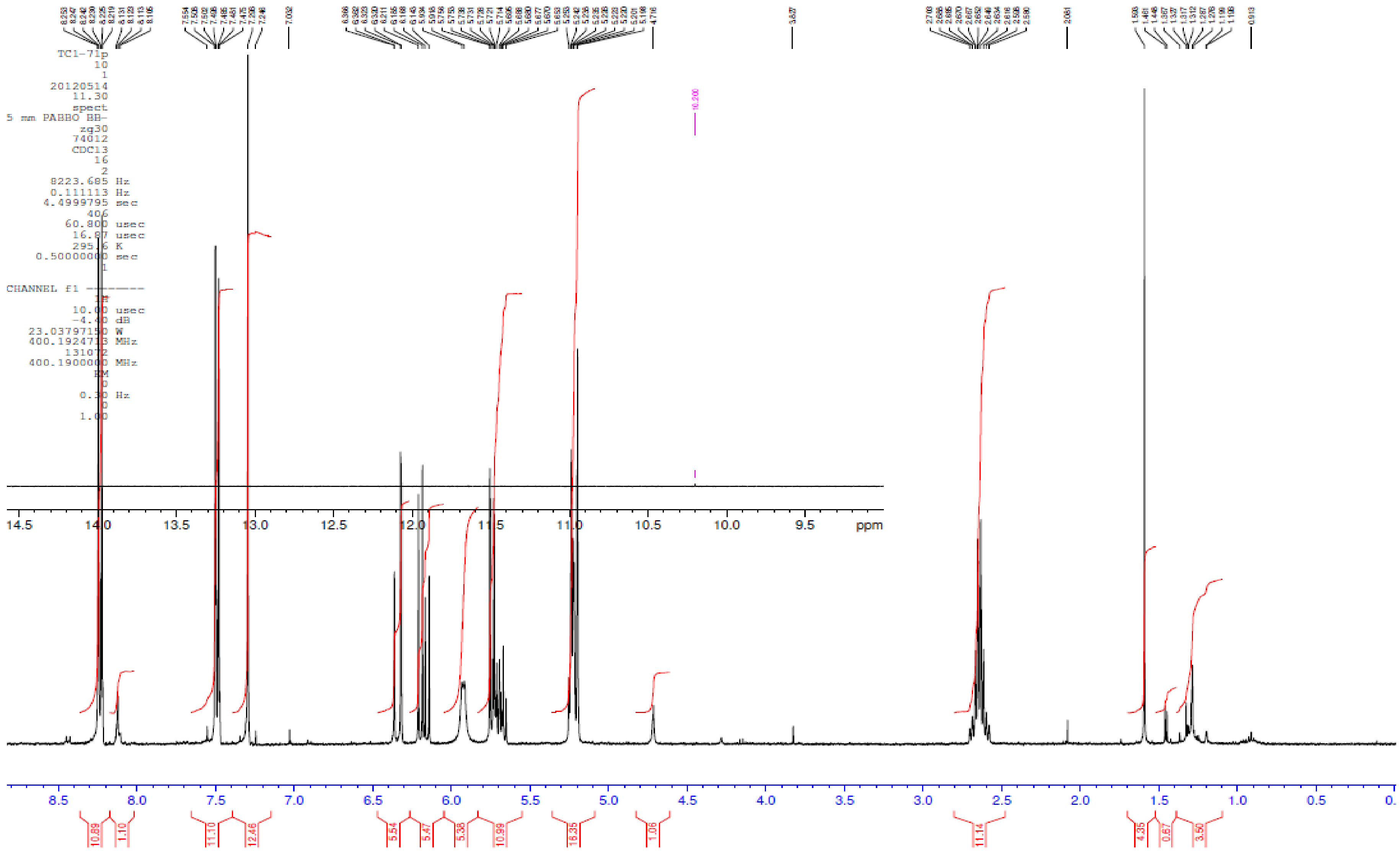

N-[1-(4-Nitrophenyl)-but-3-enyl]propenamide (8b)

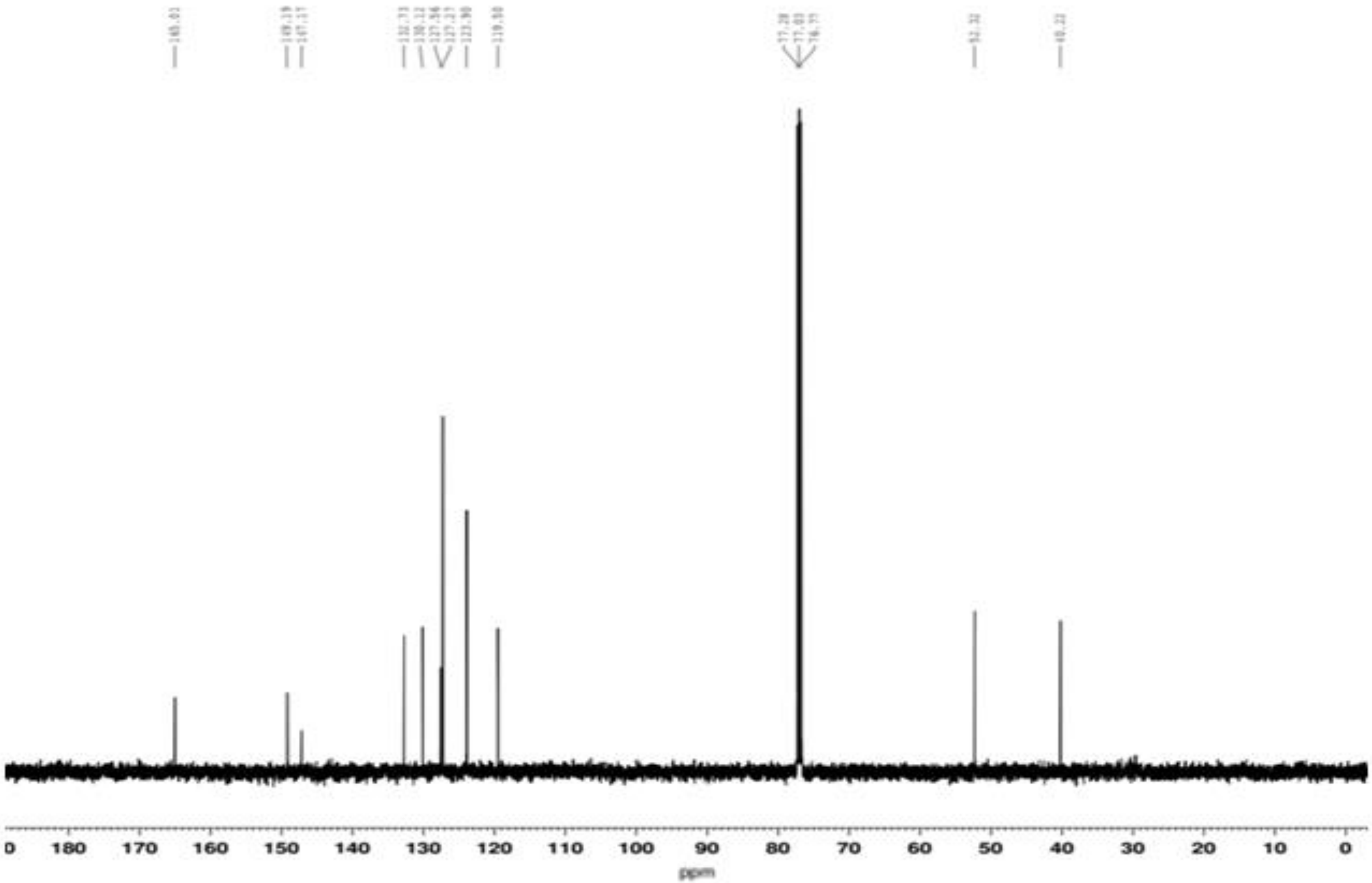

N-(1-Phenethyl-but-3-enyl)-propenamide (8c)

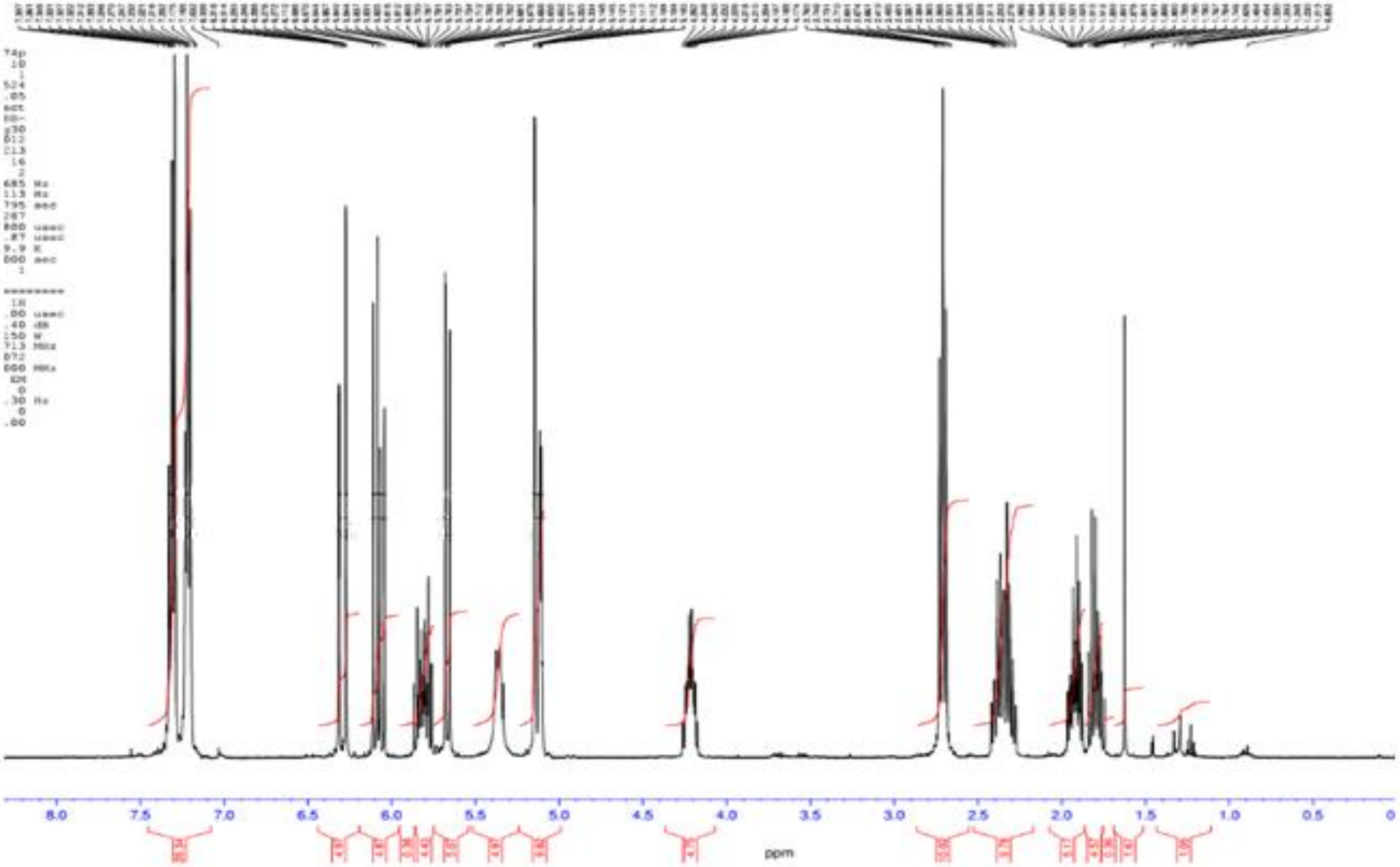

***N*-(1-Phenethyl-but-3-enyl)propenamide (8c)**

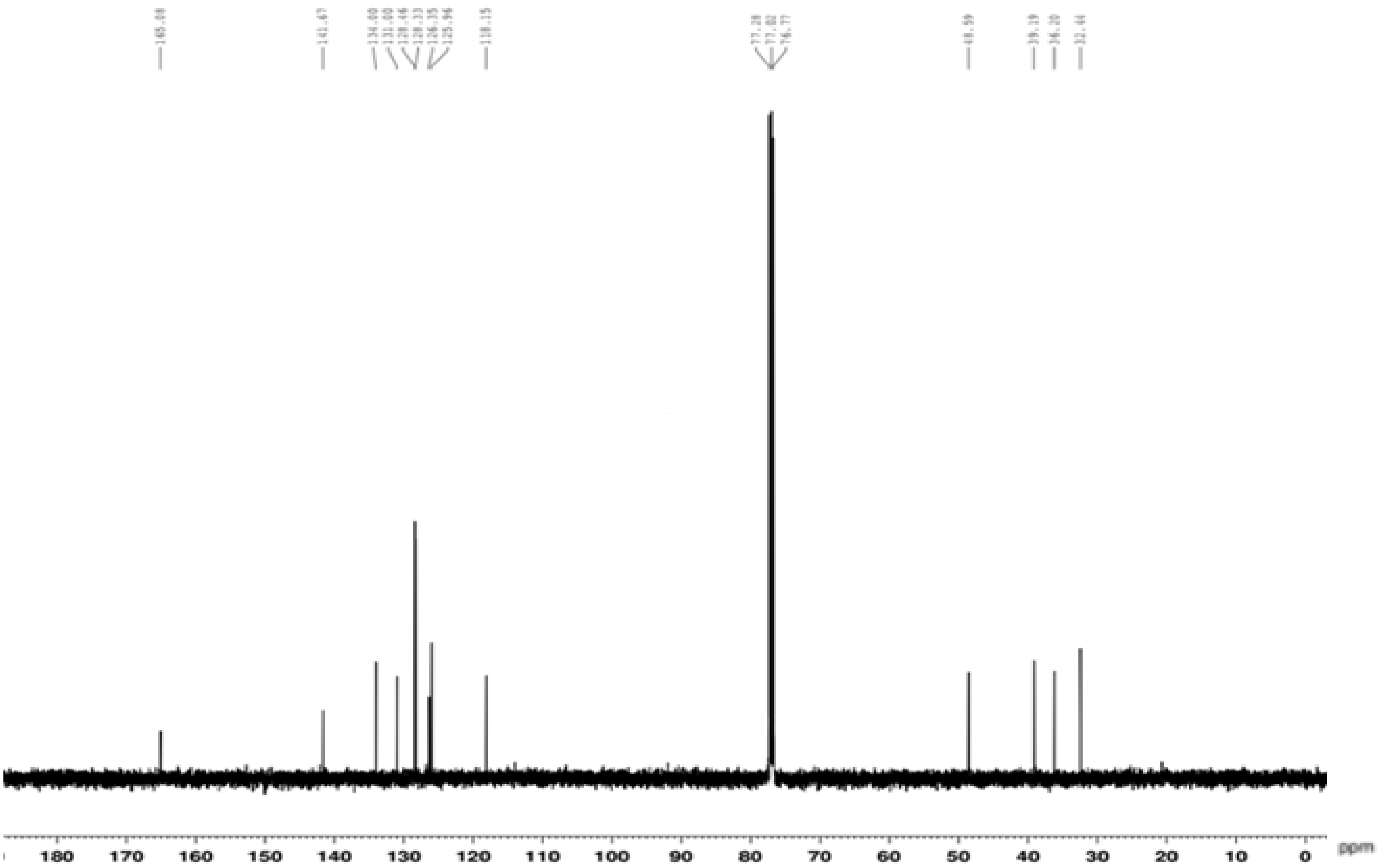

***N*-(1-Isobutyl-but-3-enyl)-propenamide (8d)**

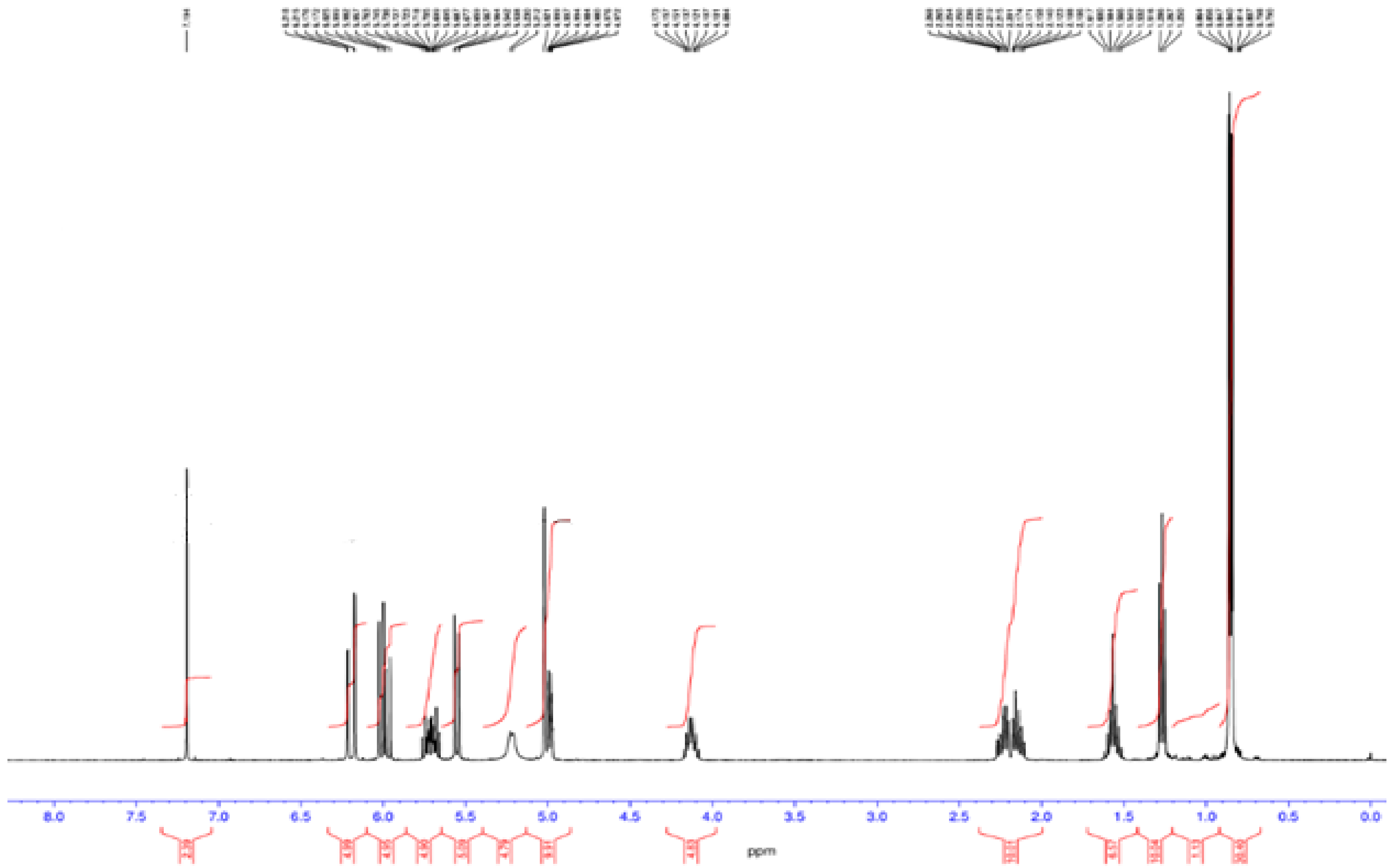

***N*-(1-Isobutyl-but-3-enyl)propenamide (8d)**

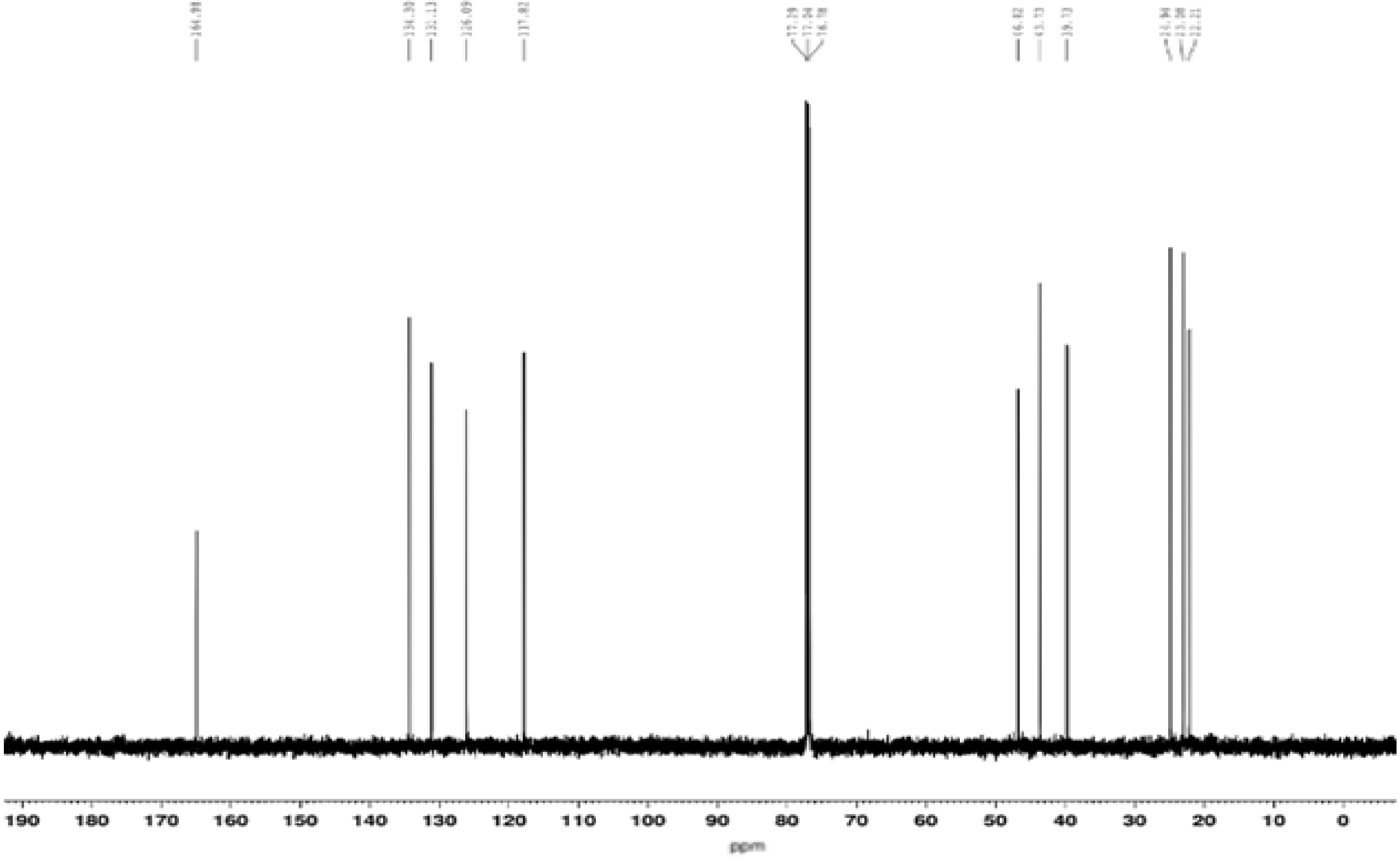

***N*-(1-Allyldecyl)propenamide (8e)**

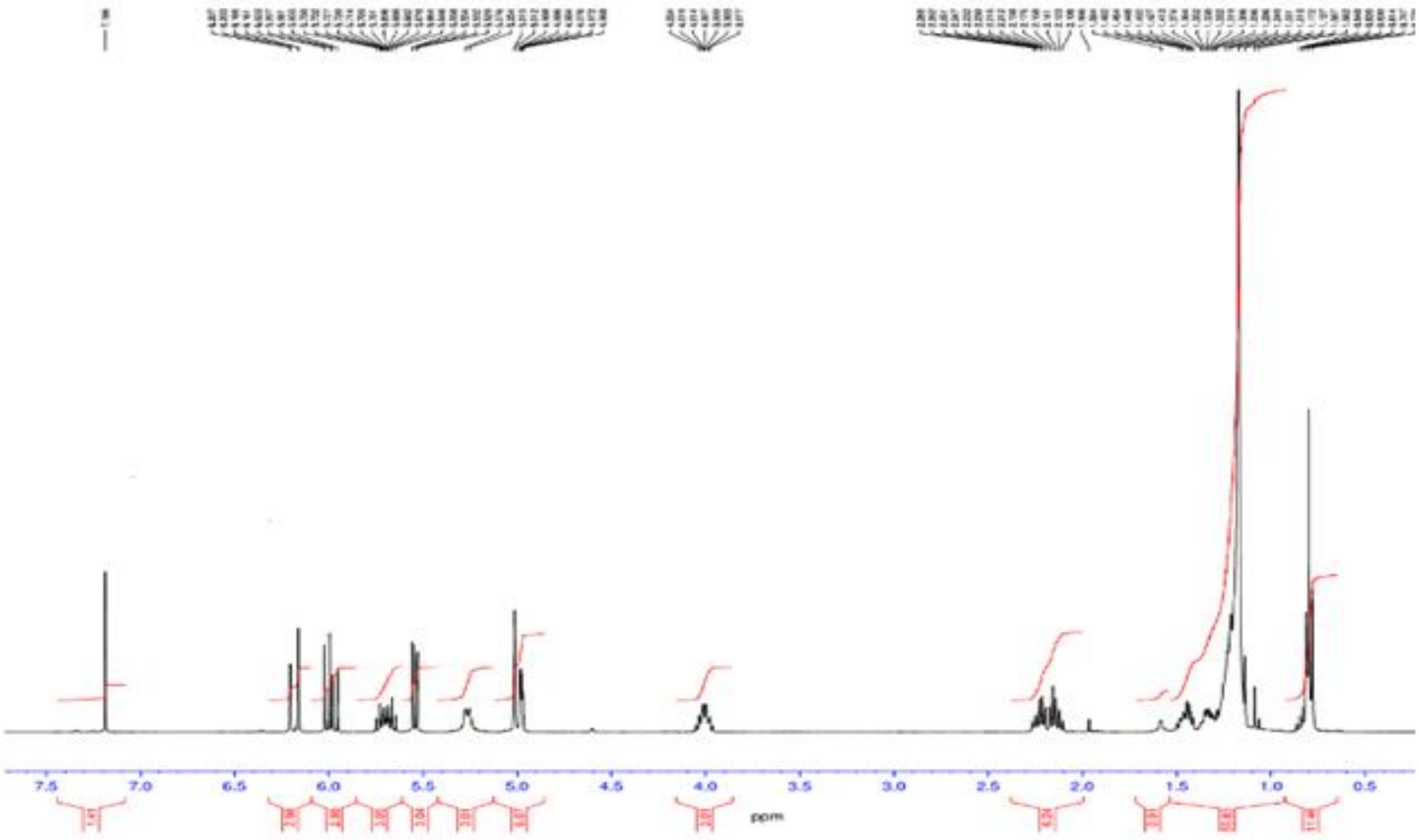

***N*-(1-Allyldecyl)propenamide (8e)**

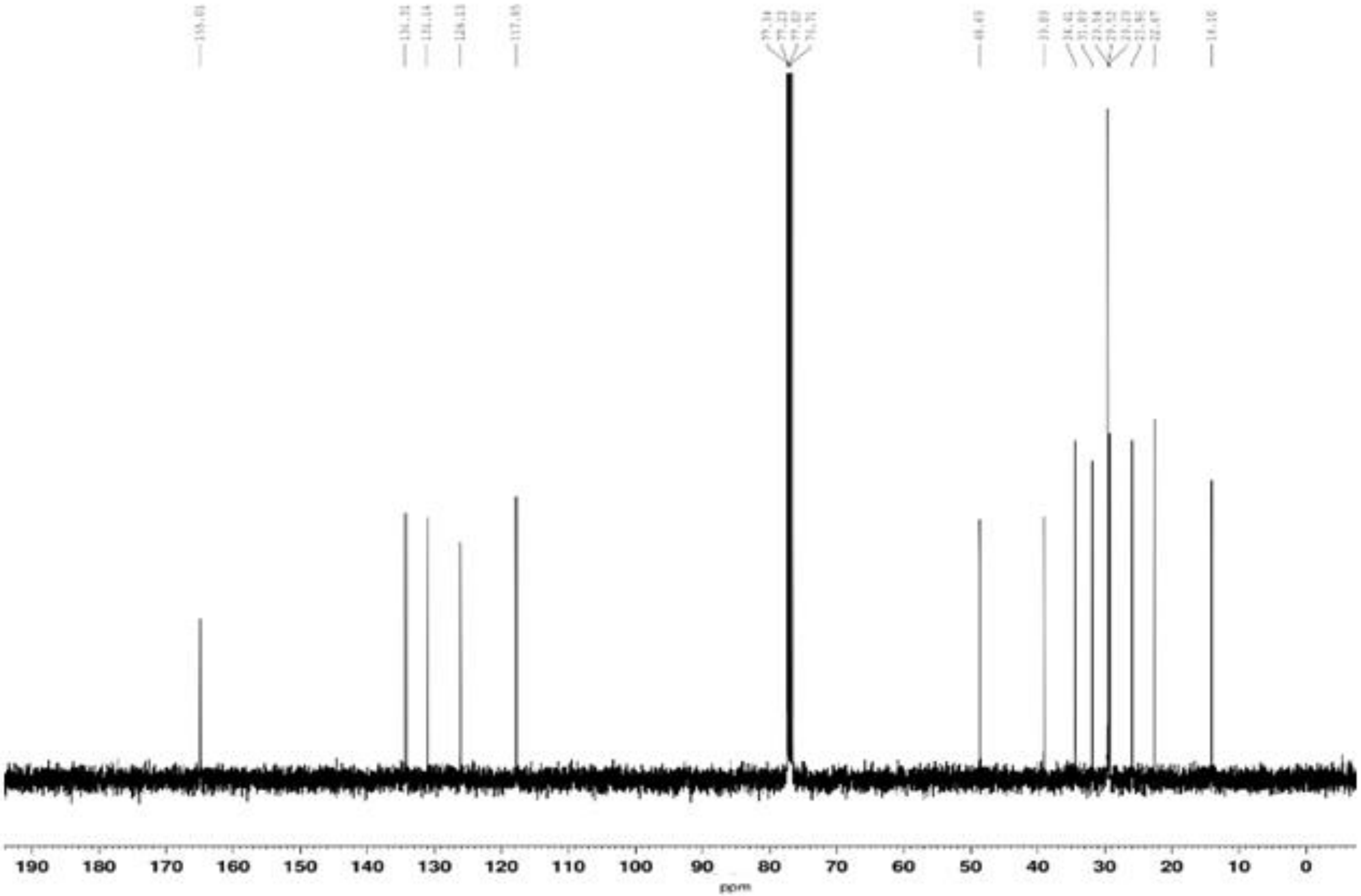

5,6-Dihydro-6-(4-methoxyphenyl)pyridin-2(1*H*)-one (9a)

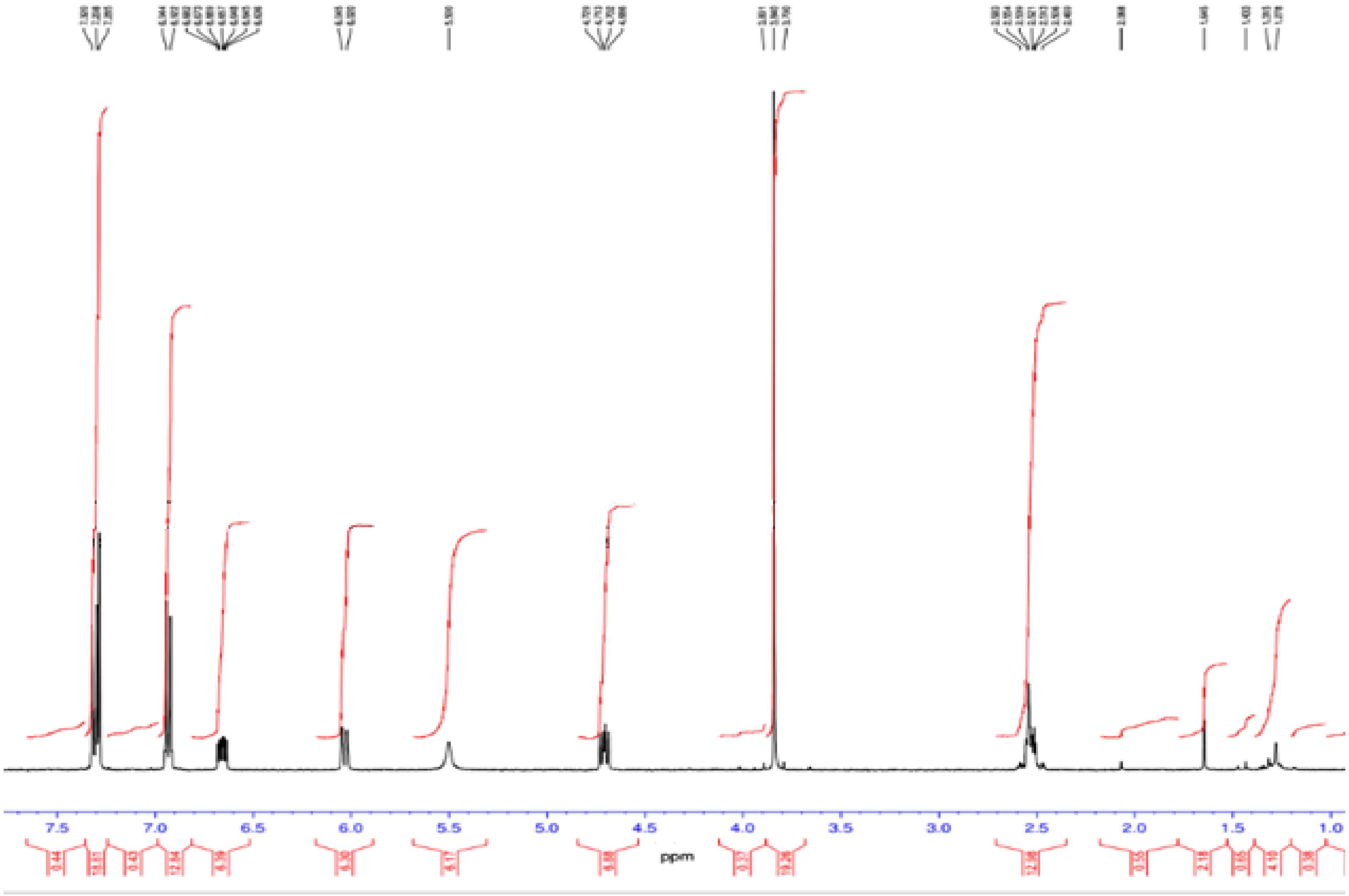

5,6-Dihydro-6-(4-methoxyphenyl)pyridin-2(1*H*)-one (9a)

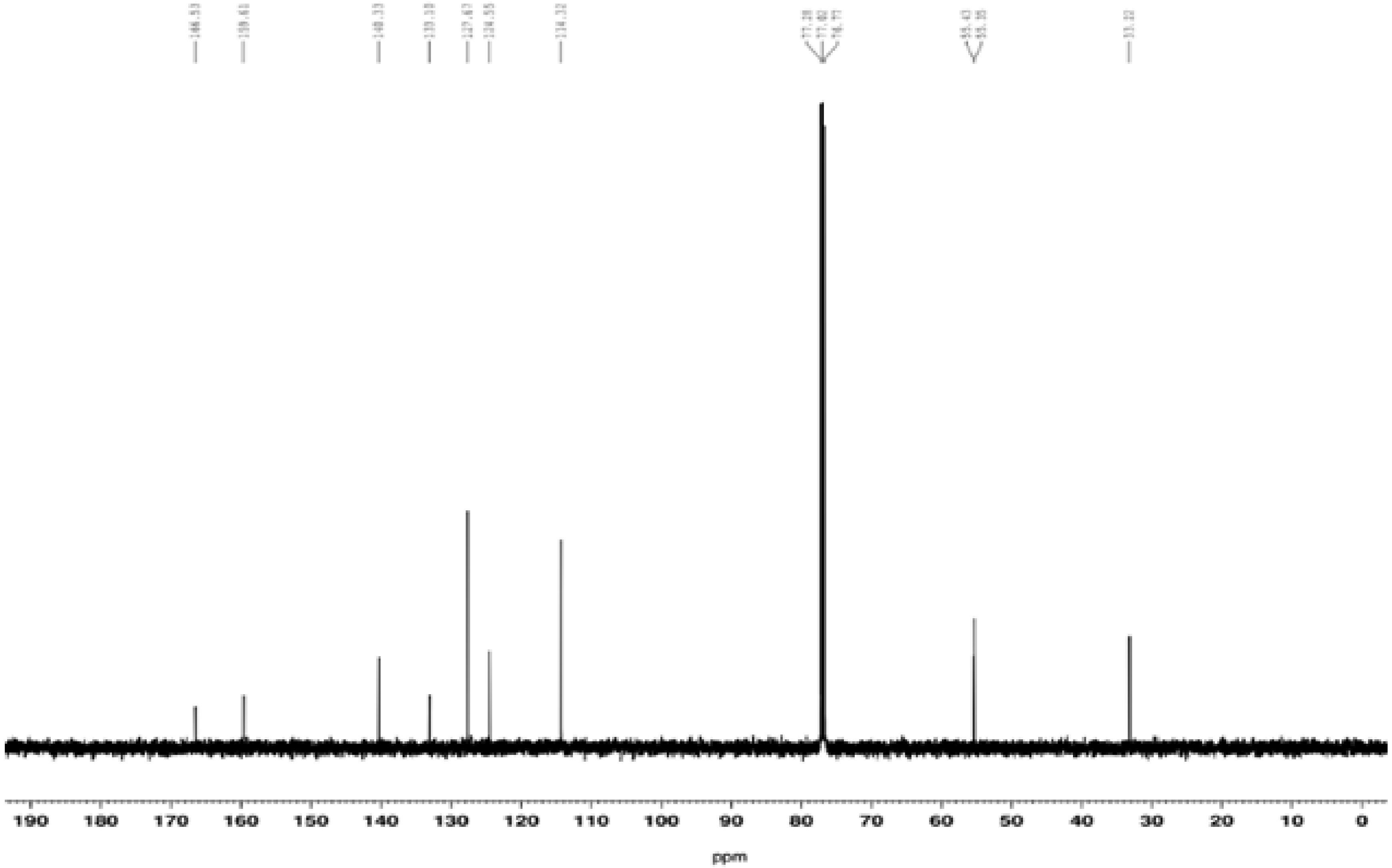

5,6-Dihydro-6-(4-nitrophenyl)pyridin-2(1*H*)-one (9b)

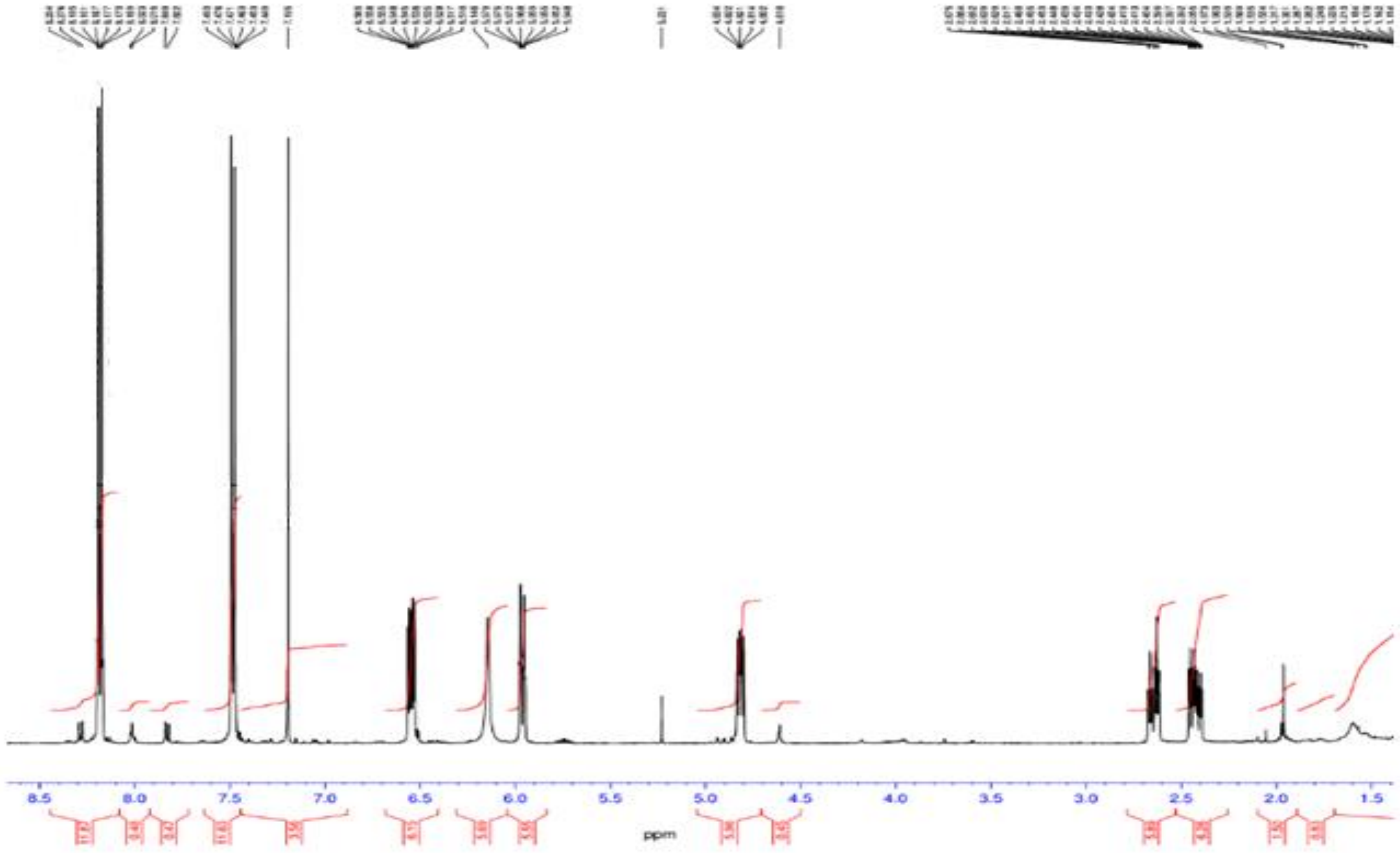

5,6-Dihydro-6-(4-nitrophenyl)pyridin-2(1*H*)-one (9b)

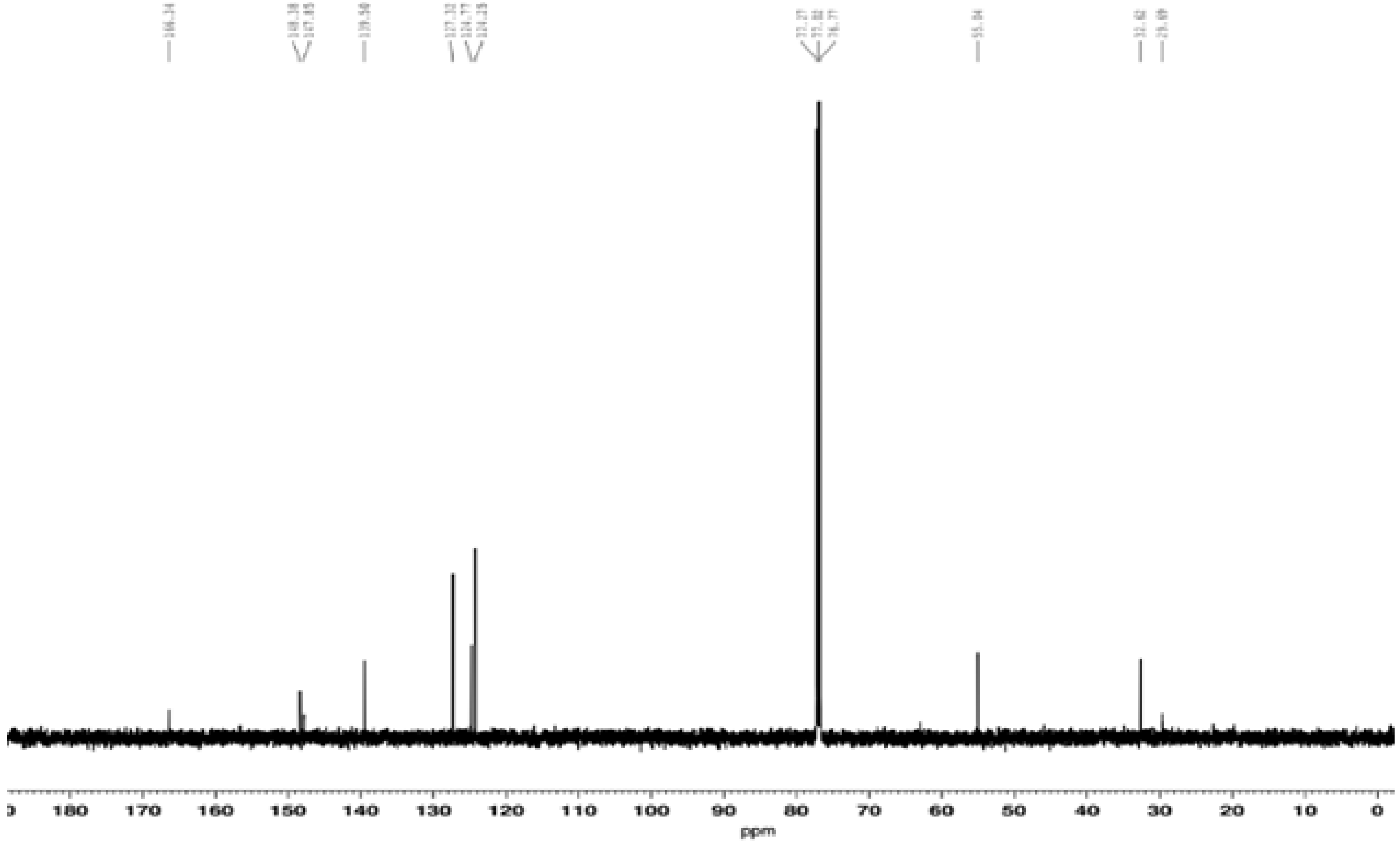

5,6-Dihydro-6-(phenethyl)pyridin-2(1*H*)-one (9c)

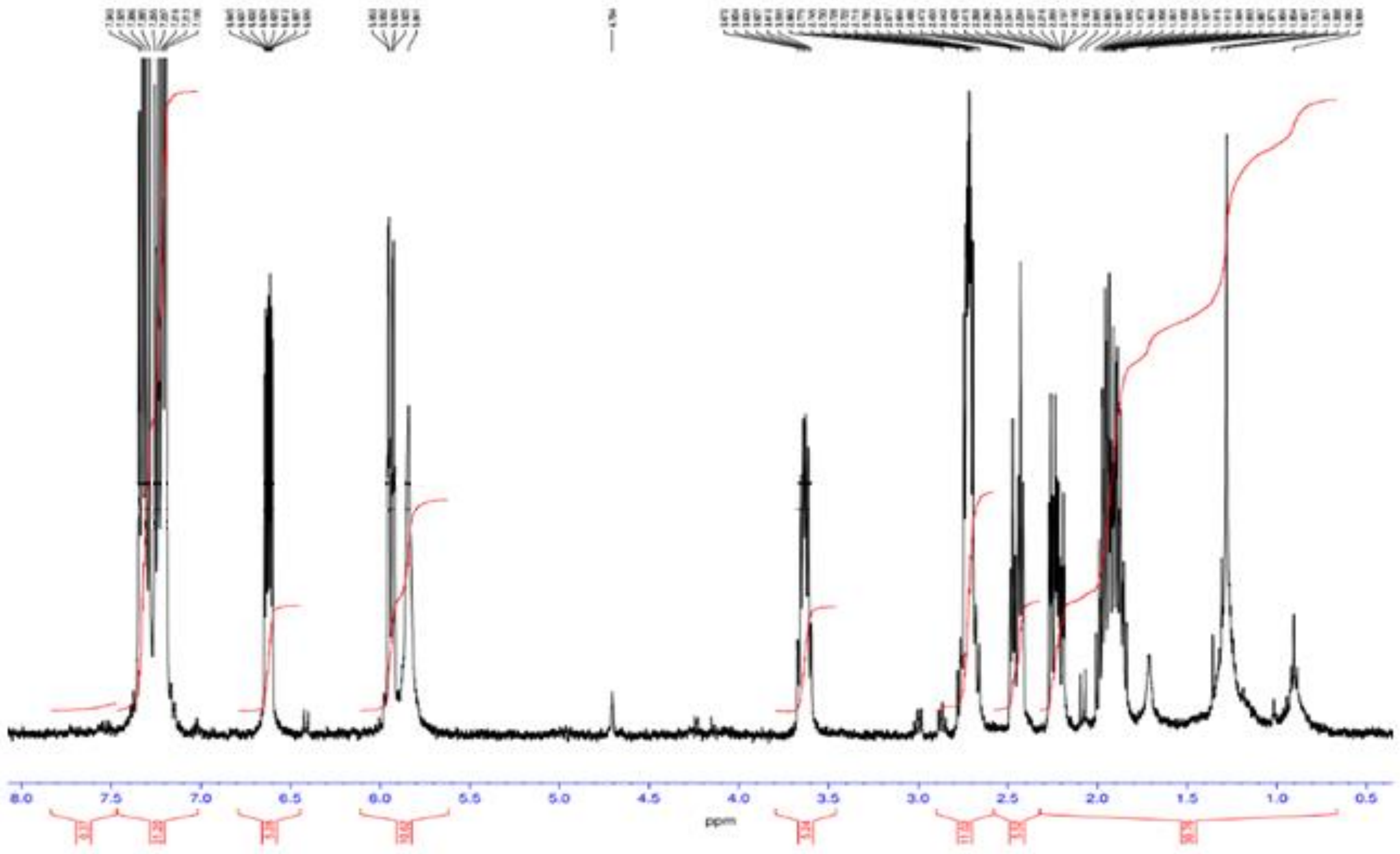

5,6-Dihydro-6-(phenethyl)pyridin-2(1*H*)-one (9c)

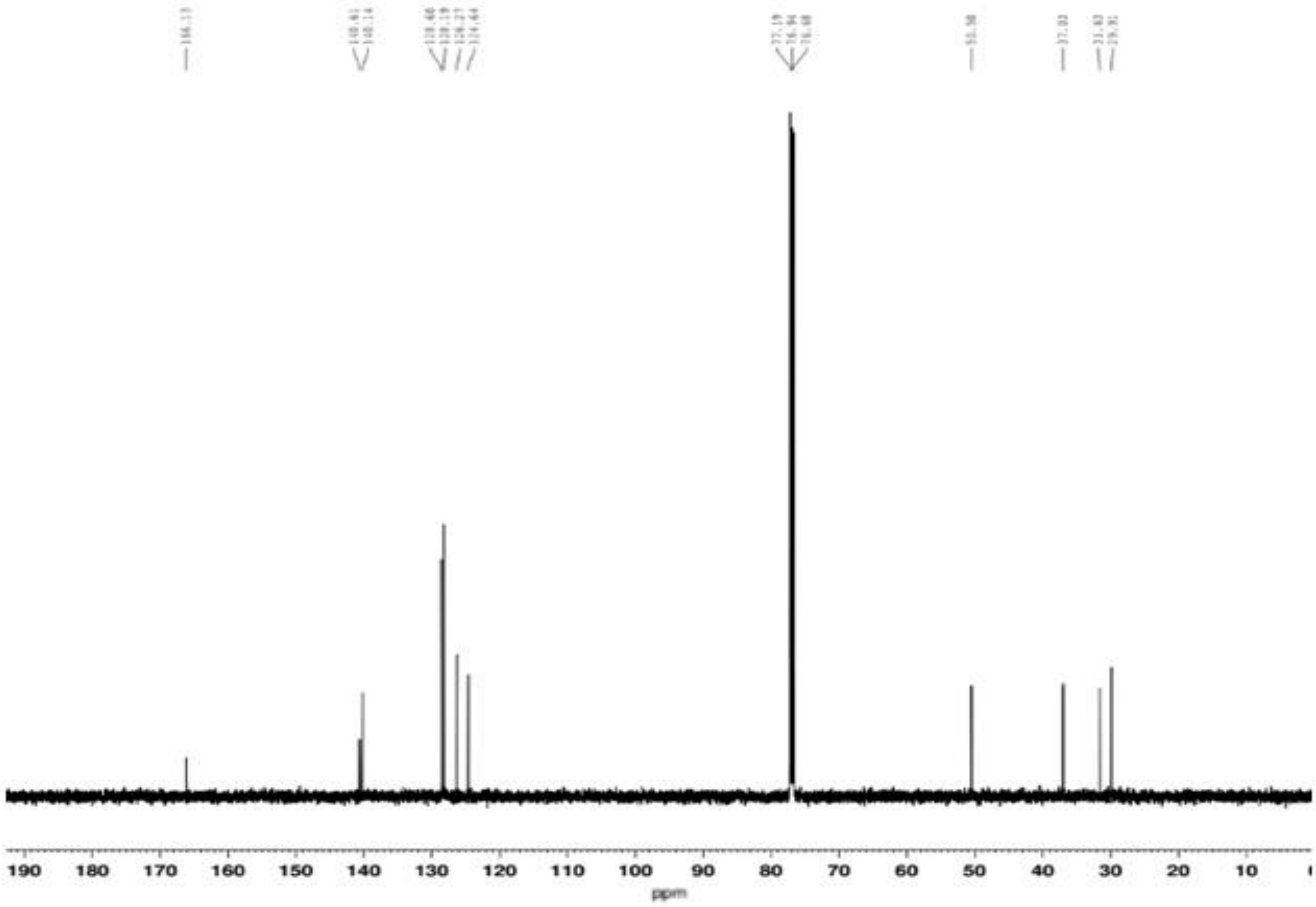

5,6-Dihydro-6-(isobutyl)pyridin-2(1*H*)-one (9d)

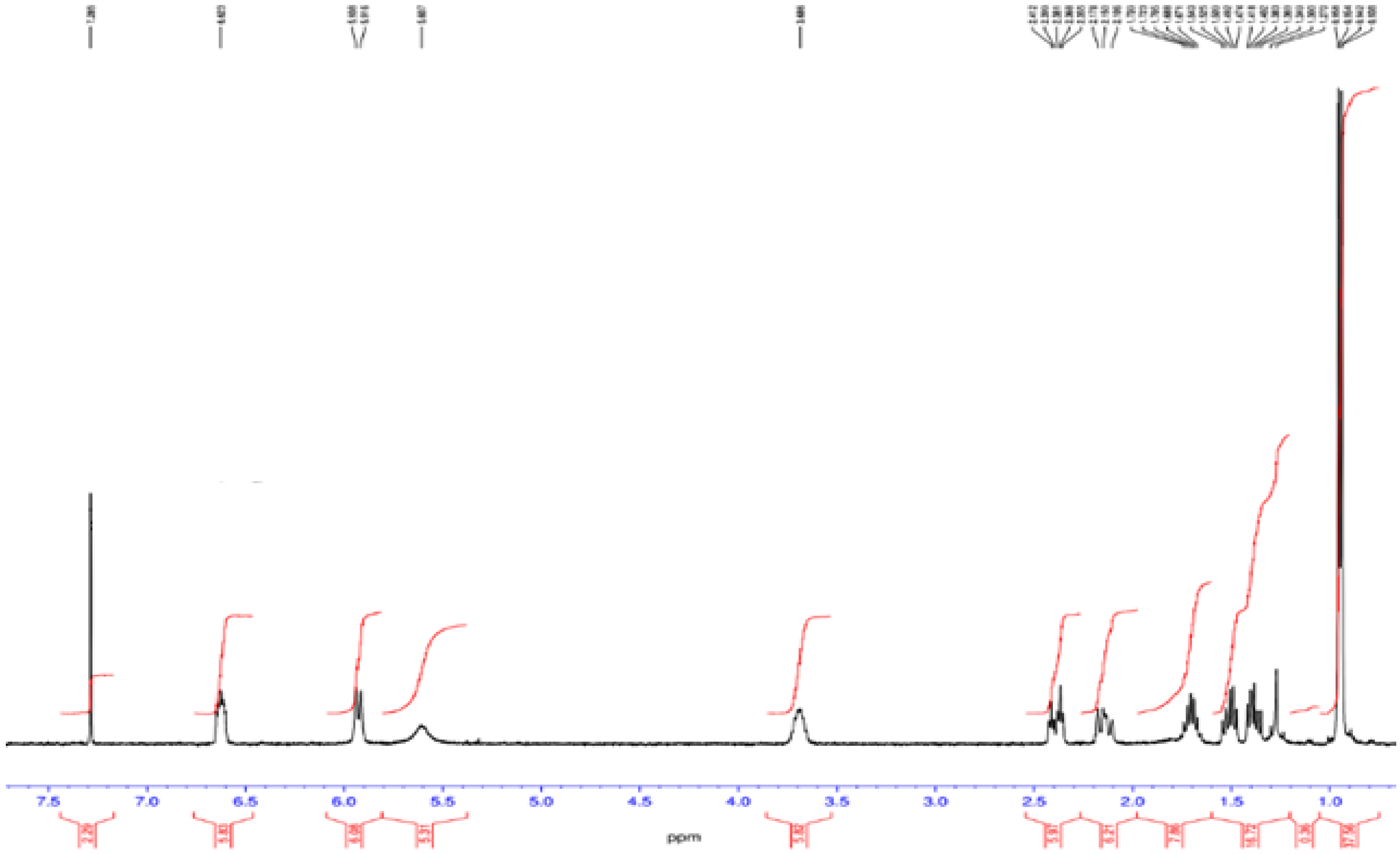

5,6-Dihydro-6-(isobutyl)pyridin-2(1*H*)-one (9d)

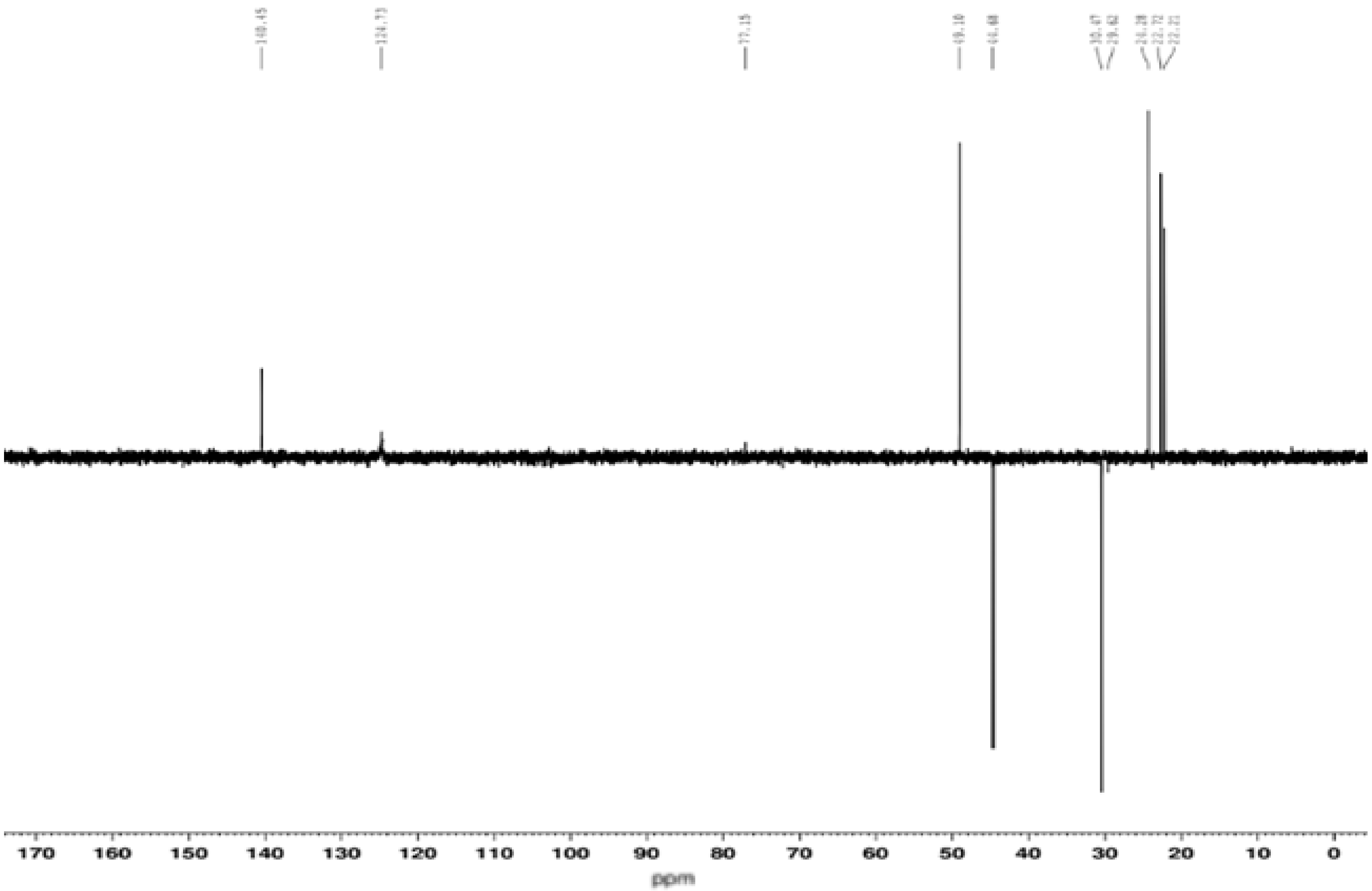

5,6-Dihydro-6-(*n*-nonyl)pyridin-2(1*H*)-one (9e)

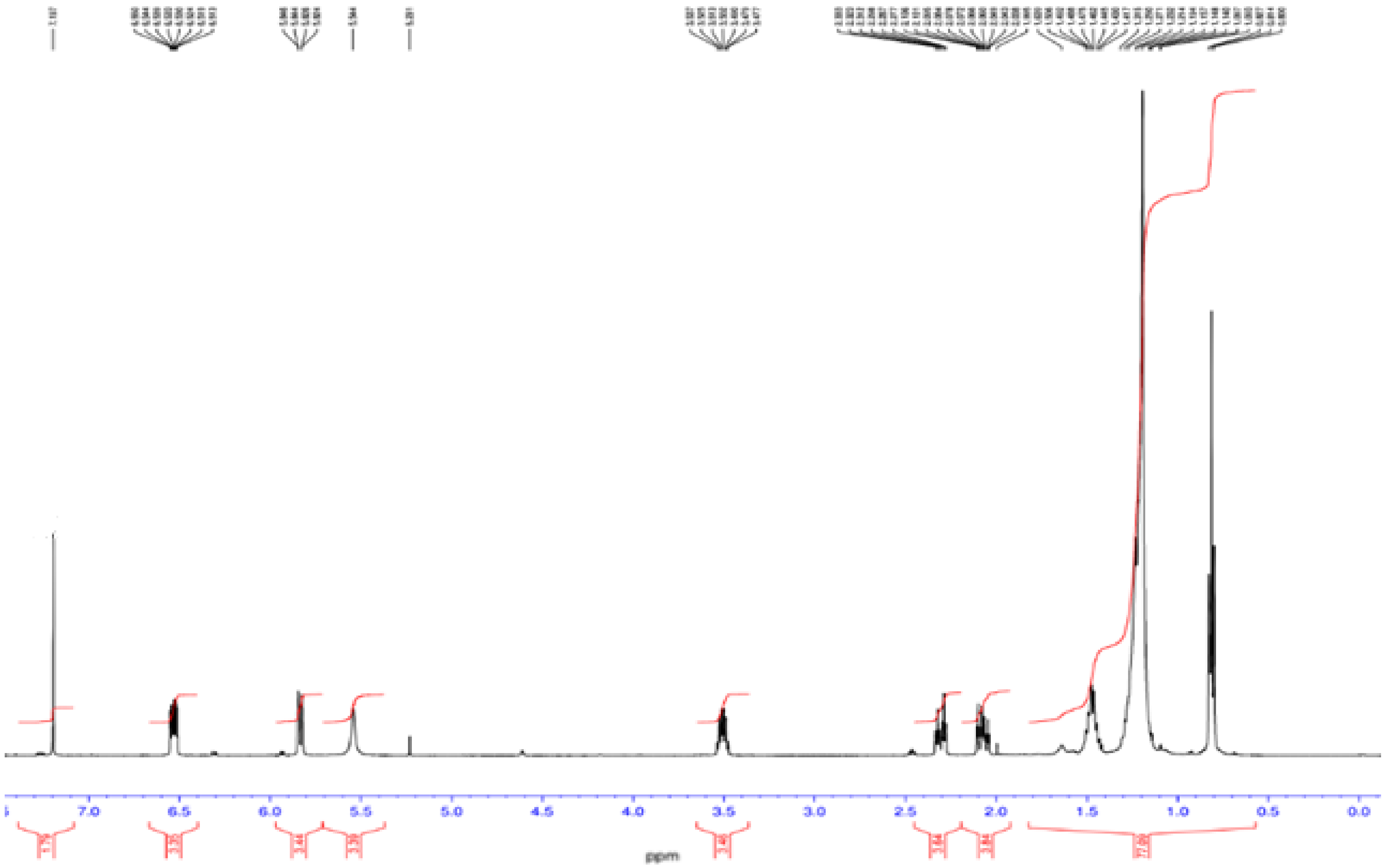

5,6-Dihydro-6-(*n*-nonyl)pyridin-2(1*H*)-one (9e)

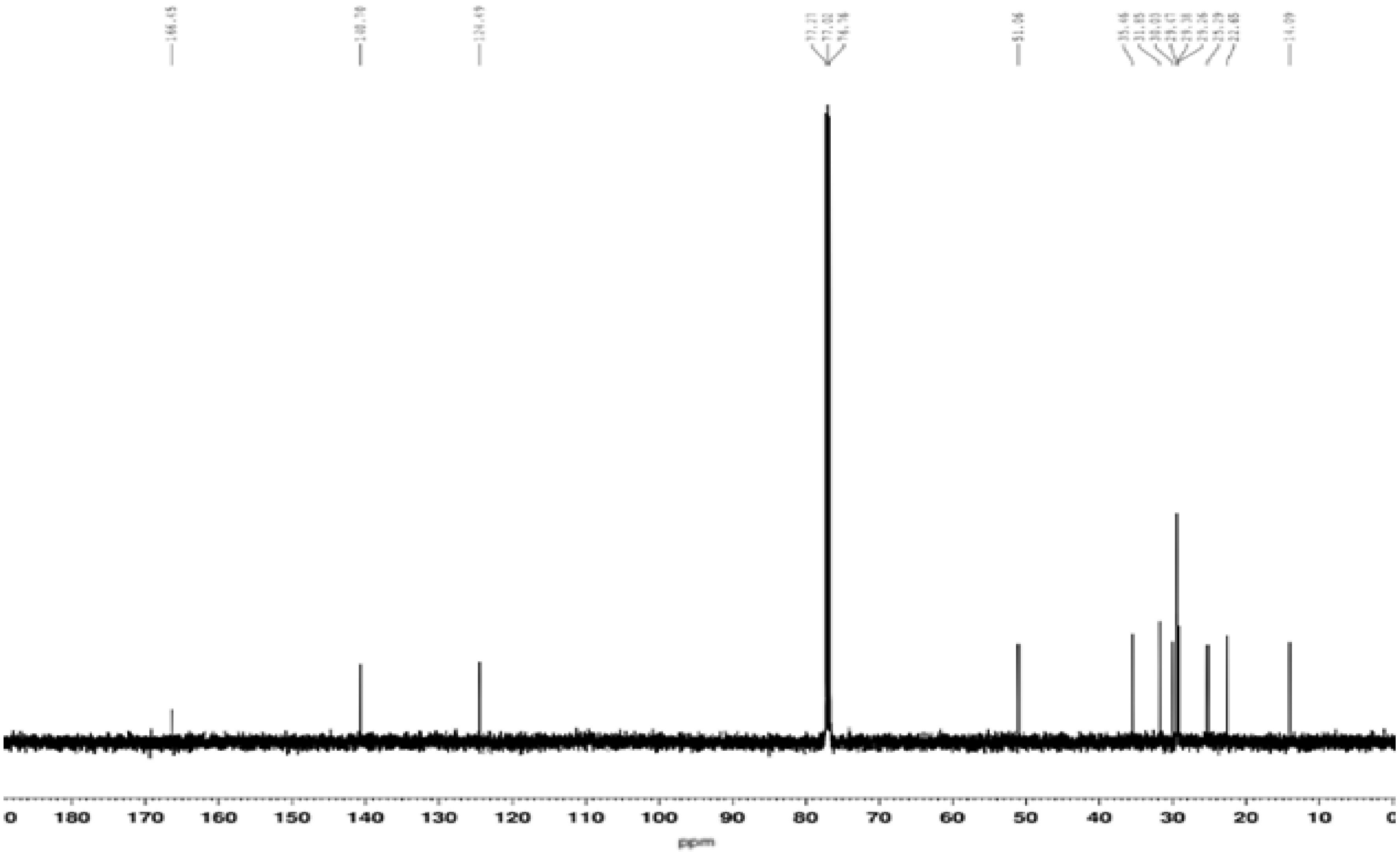

(*E*)-*N*-(1-Phenylhexa-1,5-dien-3-yl)acrylamide (10)

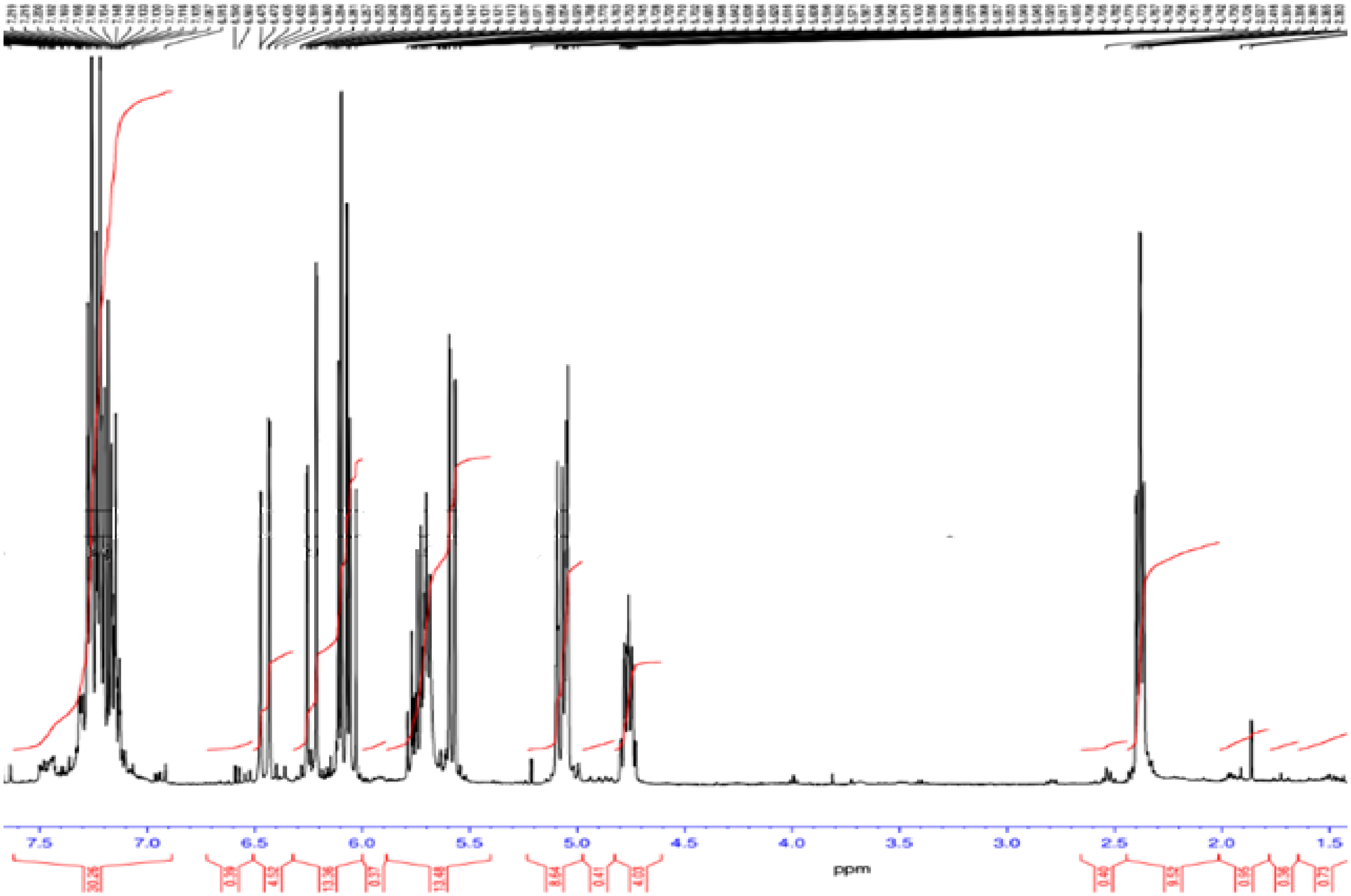

Supplement: File 1 — Experimental, characterization data and copies of spectra. [file Beilstein_J_Org_Chem-16-135-s001.pdf]
